# Tracing Ancient Migration Routes: Did *Gloydius* Pitvipers Disperse Across Paleo‐Tibet Through Temperate and Subtropical Corridors?

**DOI:** 10.1002/ece3.74238

**Published:** 2026-09-14

**Authors:** Sylvia Hofmann, Elán Pérez García, Morris Flecks, Daniel Jablonski, Chitra B. Baniya, Dennis Rödder, Joachim Schmidt

**Affiliations:** ^1^ Museum Koenig Leibniz Institute for the Analysis of Biodiversity Change Bonn Germany; ^2^ Department of Conservation Biology Helmholtz Centre for Environmental Research—UFZ Leipzig Germany; ^3^ Department of Zoology Comenius University in Bratislava Bratislava Slovakia; ^4^ Herpetological Collection, 1st Zoological Department Natural History Museum Vienna Vienna Austria; ^5^ Central Department of Botany Tribhuvan University Kathmandu Nepal; ^6^ General and Systematic Zoology, Institute of Biosciences University of Rostock Rostock Germany

## Abstract

The Himalayan–Tibetan orogen has undergone complex paleoenvironmental transformations that shaped its biodiversity, yet the timing and sequence of surface uplift—and corresponding biogeographic scenarios for endemic Himalayan taxa—remain debated. We investigated the biogeographic history of *Gloydius* pitvipers (Serpentes: Crotalinae) from the Himalaya and Hindu Kush by integrating a time‐calibrated mitochondrial phylogeny with paleoclimatic reconstructions and species distribution modeling. Our results suggest that *Gloydius* originated in East and Southeast Asia during the early Miocene (~18.6 Ma), with divergence of the Himalaya–Hindu Kush clade at ~14.5 Ma and subsequent diversification from ~11.7–6.9 Ma. Phylogenetic patterns suggest that ancestral lineages expanded westward across paleo‐Tibet via subtropical to warm‐temperate corridors, likely through a Central Paleo‐Trans‐Tibet Valley between the Gangdese and Tanggula Mountains, rather than along the Himalayan arc. A secondary corridor along the Indus–Yarlung Suture Zone may have allowed access to Himalayan transverse valleys, facilitating colonization of the western and central Himalaya, while the eastern Himalaya remained unoccupied despite suitable climate. Paleoclimatic models reveal dynamic habitat shifts over the last 3 million years, highlighting the sensitivity of these low‐dispersal taxa to climatic fluctuations. This study provides a plausible scenario for the paradoxical phylogenetic topology of Himalayan *Gloydius*, offering a biogeographic model for disjunct trans‐Tibet distributions of poorly dispersing taxa and a biotic perspective on the Miocene tectonic and environmental evolution of the Himalayan–Tibetan Orogen.

## Graphical abstract

We investigated the evolutionary and biogeographic history of Himalayan and Hindu Kush *Gloydius* pitvipers by integrating time‐calibrated phylogenetic analyses with paleoclimatic reconstructions and species distribution modeling. Our results suggest an early Miocene origin in East and Southeast Asia, followed by westward dispersal across Paleo‐Tibet via subtropical corridors and subsequent diversification within the Himalayan region. These findings provide a plausible biogeographic framework for trans‐Tibet distributions and contribute to understanding the tectonic, environmental, and evolutionary history of the Himalaya–Tibet orogen.

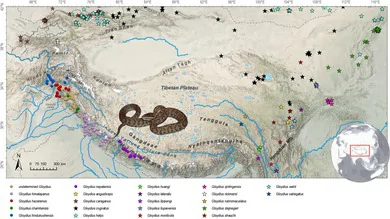

## 1 Introduction

The Himalayan‐Tibetan orogen (HTO), renowned for its geological complexity and biodiversity hotspots (Myers et al. 2000), has experienced profound paleoenvironmental transformations during its uplift history. Reconstructing the timing and nature of these changes is essential for understanding the region's climatic and ecological history and for assessing how landscape evolution has shaped the origin, distribution, and diversification of the exceptionally species‐rich biodiversity in the Himalaya and East Asia (Spicer 2017; Zhang et al. 2018; Mao et al. 2021). However, the timing, magnitude, and sequence of surface uplift in the HTO remain highly debated. Stable isotope paleoaltimetry has been widely interpreted to indicate the existence of a proto‐Tibetan Plateau at elevations of 4000–5000 m a.s.l. by the middle Oligocene (e.g., Rowley and Currie 2006; Quade et al. 2011; Xu et al. 2013; Ding et al. 2014), consistent with tectonic models proposing a high‐elevation Paleogene Tibet (Murphy et al. 1997; Tapponnier et al. 2001; Mulch and Chamberlain 2006; Kapp et al. 2007; Fang et al. 2020) and implying the existence of extensive alpine environments by that time. In contrast, the fossil record provides no evidence for alpine biota prior to the late Miocene. Instead, fossil assemblages indicate that tropical and warm‐temperate ecosystems remained widespread across much of the region until the early Miocene (Zhou et al. 2007; Sun et al. 2014; Wei et al. 2016; Wu et al. 2017; Wang et al. 2018; Ai et al. 2019), revealing a fundamental discrepancy between isotope paleoaltimetric and paleontological evidence.

Although traditional methods, such as sedimentary records, isotopic analyses, and paleontological data, have significantly contributed to our knowledge of the HTO's evolution, they still provide inconsistent insights. To complement these approaches, a promising yet underexplored approach lies in examining the seemingly paradoxical phylogenies of Himalayan species groups that have evolved within this dynamic landscape. Such phylogenies reveal unexpected evolutionary relationships that challenge conventional models of Himalayan species' historical biogeography and the associated paleoenvironmental scenarios. Specifically, these phylogenies often show an intriguing pattern: taxa from the far western Himalaya represent ancient lineages or are more closely related to those from the eastern margin of the Tibetan Plateau, thousands of kilometers away, rather than to geographically proximate taxa in the Central Himalaya. This surprising finding—observed in groups such as Himalayan ground beetles (Schmidt et al. 2012) and amphibians (Hofmann et al. 2021; Hofmann, Podsiadlowski, et al. 2024)—contradicts the widely accepted view that species distribution follows a stepwise migration pattern from east to west (or vice versa), a framework often used to explain the colonization and evolution of Himalayan biodiversity. These unexpected phylogenetic relationships indicate that the region's evolutionary history is much more complex than previously thought, offering new insights into the processes shaping the biodiversity of the Himalaya and surrounding areas. The HTO, therefore, presents a unique opportunity to explore how species have responded to changing paleoenvironmental conditions, providing a clearer understanding of the Paleogene Tibetan landscape and its topography.

**FIGURE 1:**
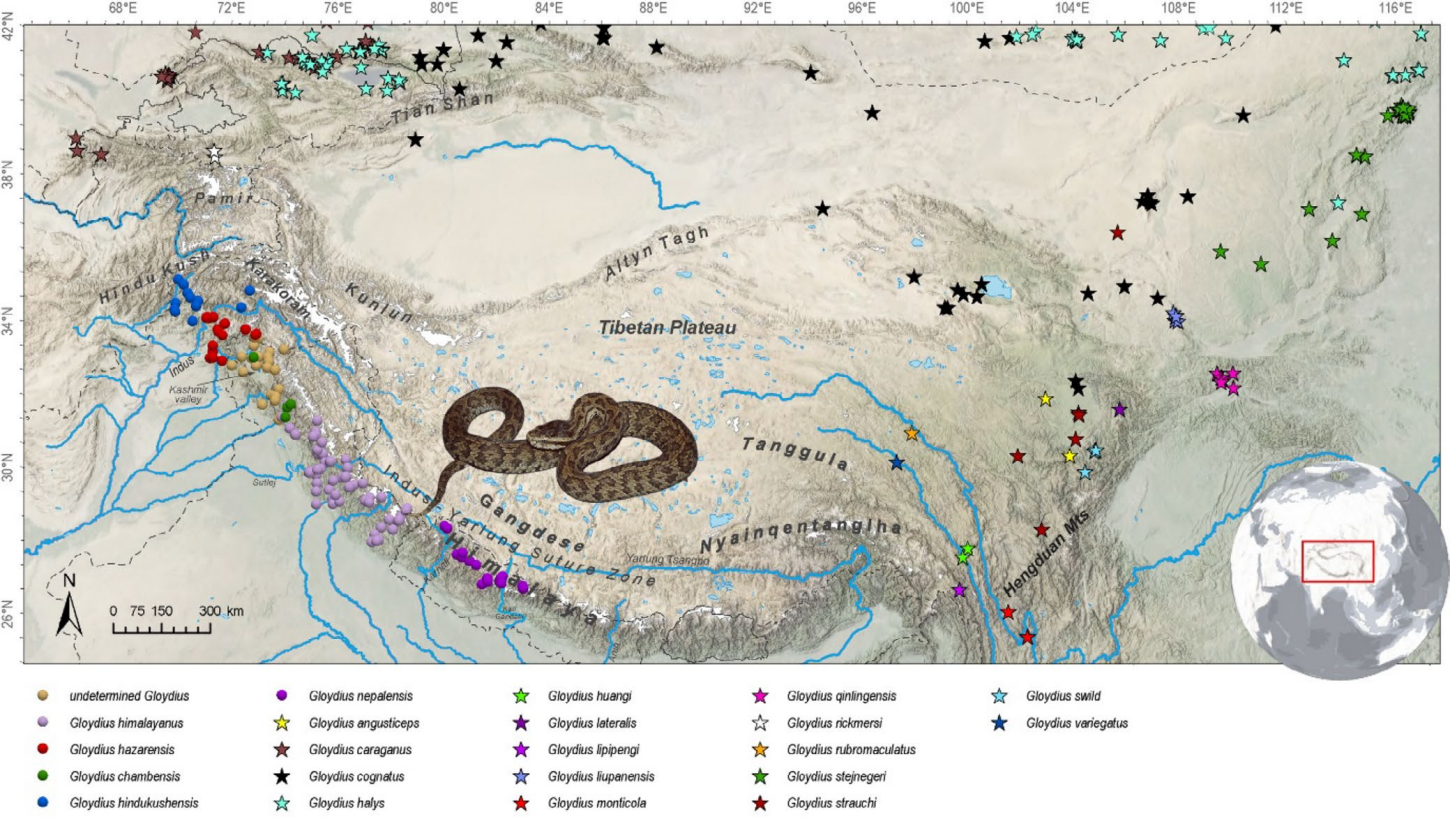
Geographic distribution of recognized *Gloydius* species distributed in the Himalaya and Hindu Kush (colored dots), together with records of related *Gloydius* species from adjacent regions of the Himalaya‐Tibet orogen (colored stars). Records labeled as “undetermined” species were originally assigned to *G. himalayanus* but may include individuals of *G. chambensis* or *G. hazarensis*. The inset photograph shows *G. nepalensis* ; photo credit: Frank Tillack. Coordinates of records from the Himalayan arc are provided in Table S1. Records of other *Gloydius* species were obtained from Global Biodiversity Information Facility (GBIF; https://www.gbif.org).

Recently, pitvipers (Crotalinae) of the genus *Gloydius* have emerged as promising models for biogeographic research in the Himalaya and Hindu Kush—two of Asia's least explored mountain systems. A comprehensive taxonomic revision of the *G. himalayanus* complex uncovered several deeply divergent, geographically structured lineages with restricted ranges, highlighting their potential for reconstructing historical patterns of isolation and dispersal (Jablonski et al. 2026) (Figure 1). By integrating dated phylogenetic data with paleoclimatic reconstructions, this study provides new insights into the origin and historical biogeography of these taxa and the paleoenvironmental evolution of the HTO.

## 2 Material and Methods

We used a recently published mitochondrial sequence dataset of *Gloydius* species from the Himalaya and Hindu Kush which comprised sequences of 12S, 16S, cytochrome *b* (cyt*b*) and NADH dehydrogenase 4 (nd4) gene regions (Jablonski et al. 2026). Additional sequence data of all currently recognized species of the genus, as well as data of appropriate outgroups corresponding to the target loci were retrieved from GenBank (taxon sampling, specimen information, and sequence accession numbers for each species are provided in Table S2), aligned with MEGA11 (Tamura et al. 2021) and concatenated. Sampling intensity varied among species because sequence availability differed among taxa. Whenever possible, multiple individuals were included to capture intraspecific variation, whereas taxa represented by few specimens were retained to maximize taxonomic coverage. The final alignment contained 179 taxa (2693 bp: 12S 408 bp; 16S 482 bp; cyt*b* 1125 bp; nd4 678 bp). Based on this locus partitioned dataset, a maximum likelihood (ML) phylogeny was inferred with IQ‐TREE2 v2.1.2 (Minh et al. 2020) using the partition scheme and substitution models as selected by ModelFinder (Kalyaanamoorthy et al. 2017) and PartitionFinder (Lanfear et al. 2012) implemented in IQ‐TREE 2, and 100,000 bootstrap replicates.

### 2.1 Divergence Time Estimation

For estimating divergence dates we used BEAST2 v.2.7 (Bouckaert et al. 2019) and the concatenated mtDNA dataset. We imposed the following 11 secondary time constraints from the fossil calibrated Viperinae phylogeny of Šmíd and Tolley (2019). All constraints were implemented through normally distributed priors: (i) MRCA (Most Recent Common Ancestor) of Azemiopinae and Crotalinae: 31.6 Ma, range 26.9–36.3; (ii) MRCA of *Protobothrops*, *Gloydius, Agkistrodon, Crotalus*, and *Sistrurus*: 24.7 Ma, range 21.0–28.4; (iii) MRCA of *Agkistrodon*, *Crotalus*, and *Sistrurus*: 20.2 Ma, range 16.9–23.5; (iv) MRCA of *Crotalus* and *Sistrurus*: 13.3 Ma, range 10.8–15.9; (v) MRCA of *Macrovipera* and *Montivipera*: 12.6 Ma. range 11.7–13.5; (vi) MRCA of *Daboia mauritanica* and *D. russelii* split: 16.11 Ma range 13.6–18.7; (vii) separation of *Vipera ammodytes* from all other *Vipera* in the tree: 13.4 Ma, range 11.5–15.3; (viii) MRCA of *Vipera aspis* and *V. latastei* split: 7.1 Ma, range 5.6–8.7; (ix) MRCA of *Vipera berus*, *V. seoanei* , *V. berus walser, V. kaznakovi, V. graeca, V. ursinii, V. eriwanensis, V. lotievi*, and *V. renardi* : 6.0 Ma, range 5.1–6.9; (x) MRCA of *Vipera berus* and *V. seoanei* : 4.7 Ma, range 3.8–5.7; (xi) MRCA of *Vipera ursinii* and *V. graeca* : 2.6 Ma, range 2.0–3.2.

We performed three independent BEAST2 runs, defining a chain length of 10 million for each run, a thinning interval of 1000, a lognormal relaxed clock model, a birth‐death tree prior, and a random starting tree. Substitution models were inferred using bModelTest (Bouckaert and Drummond 2017). Runs were then combined with BEAST2 LogCombiner v.2.6.7. Convergence and stationarity were assessed by confirming an average standard deviation of split frequencies < 0.01 and effective sample size (ESS) values > 200 for all parameters. ESS values and parameter traces were examined in Tracer. The final tree was generated with TreeAnnotator v.2.6.2 and visualized with FigTree v.1.4 (Drummond and Rambaut 2007).

### 2.2 Species Distribution Modeling

To assess the potential geographic distribution of the Himalayan *Gloydius* species (*G. hazarensis*, *G. himalayanus* , *G. hindukushensis*, and *G. nepalensis* ), we computed species distribution models (SDMs). The models were computed based on the species' currently realized climatic niches and subsequently projected onto paleoclimatic scenarios. Our species distribution modeling approach relies on a static characterization of the species' realized niches derived from present‐day taxon occurrence data. Species locality records associated with geographical coordinates were collected from our previous study (Jablonski et al. 2026), in which all available occurrence records were critically evaluated and assigned to the appropriate species based on an integrative assessment of morphology, molecular evidence, type material, and geographic information. The curated dataset used for ecological niche modeling is listed in Table S1. In some cases, a clear distinction between the records of *G. himalayanus* and *G. chambensis* could not be made due to the potential overlap in their geographic distributions and their significant morphological similarity (Jablonski et al. 2026). Therefore, it could not be excluded that some records identified as *G. himalayanus* might correspond to *G. chambensis* and vice versa. Hence, only unambiguously identified records of *G. himalayanus* were used and *G. chambensis* was omitted from modeling due to the limited availability of clearly identified records.

As environmental layers, we used a set of bioclimatic variables for the present time (1979–2013) from the CHELSA database v.2.1 (Karger et al. 2017) at a spatial resolution of 2.5 arc minutes. Collinearity between variables may result in model overfitting. To account for this potential error, we selected only one predictor among pairs with a Spearman's pairwise rank correlation of higher than |0.75| focusing on predictors reflecting thermal energy and water availability as expected to be relevant for an ectothermic organism (i.e., annual mean temperature = bio1, annual precipitation = bio12, precipitation of the driest month = bio14, precipitation seasonality = bio15, precipitation of the warmest quarter = bio18, and precipitation of the coldest quarter = bio19). Corresponding paleoclimatic variables for the mid‐Pliocene warm period (mPWP v1.0, 3.205 Ma) (Hill 2015), and mid‐Pliocene cooling period (Marine Isotope Stage M2 v1.0, 3.3 Ma) (Dolan et al. 2015) were downloaded with a spatial resolution of 2.5 arc min from the paleoclimate database (Brown et al. 2018). Because suitable paleoclimatic layers for species distribution modeling were unavailable for the Miocene, projections were restricted to the oldest available paleoclimatic scenarios (mPWP and M2).

Currently realized niches of each species were compared using density kernel estimates, as computed with the sm.density.compare function of the sm (Bowman and Azzalini 2021) package for R.

For SDM calculation and model fitting, we used MaxEnt ver. 3.4.4 (Phillips and Dudík 2008; Phillips, Anderson, et al. 2017; Phillips, Dudík, and Schapire 2017) using a model optimization procedure based on AICc as described in (Ginal et al. 2022) and the relevant functions of the ENMeval package for R (Kass et al. 2021). For each species, individual background points were selected as defined by a radial buffer of 0.5° enclosing the respective record, which was further clipped to the respective WWF biomes. Furthermore, only one species record per 2.5 arc min grid cell was used for model training. For further details of the analysis pipeline, we refer to the supporting information of Noori et al. (2025). We tested all possible combinations of regularization parameters (0.5–5 in steps of 0.1) and feature classes each 100 times using a data split of 80% of the records used for training and 20% for model testing using the area under the receiver operating characteristic curve (AUC). For the final models, we selected those settings with an average AUC > 0.7 and the lowest AICc and used the same data split strategy. In addition to AUC, we computed the average True Skill Statistic (TSS) across all replicates to assess model performance (Allouche et al. 2006). As a lower threshold, we selected the average 10% training omission. Potential areas with non‐analogous climatic conditions exceeding the training range of the models were identified using multivariate environmental similarity surfaces (Elith et al. 2010).

## 3 Results

### 3.1 Phylogenetic Relationships and Divergence Times in *Gloydius*

**FIGURE 2:**
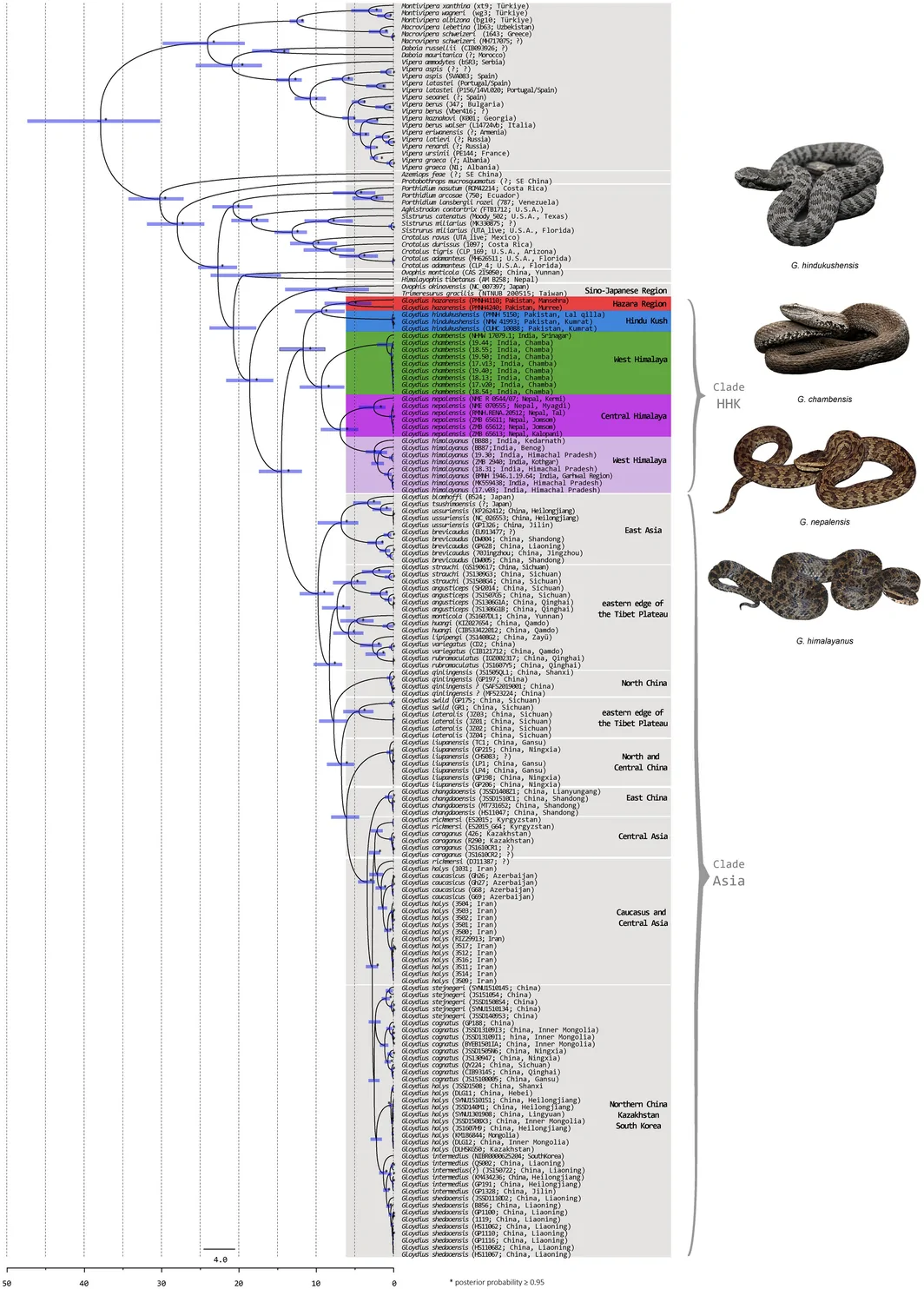
Time‐calibrated phylogeny of *Gloydius* with selected outgroups. Divergence times were inferred in BEAST2 using a relaxed molecular clock model. Tip labels include the species name, followed (in brackets) by the associated voucher, isolate, field, or GenBank accession number, and locality information (country and, where available, more specific locality details). Horizontal bars at nodes indicate 95% highest posterior density (HPD) intervals. Nodes with posterior probability support ≥ 0.95 are marked with an asterisk. Himalayan clades are color‐coded according to the geographic map (Figure 1). Photo credit: Frank Tillack ( *G. nepalensis* , *G. himalayanus* ) and Daniel Jablonski (*G. hazarensis*, *G. hindukushensis*). A high‐resolution version is available as Figure S2.

We provided a dated phylogeny for the genus *Gloydius*, comprising all currently recognized species including those of the Himalaya and Hindu Kush (HHK) clade. The topologies of both BI and ML phylogenetic trees were largely consistent, with full concordance observed for the HHK lineages and their sister clades (Figure 2, Figure S1). Most divergences were strongly supported. Higher‐level nodes showed a sister relationship between *Gloydius* and the East Asian pitvipers *Ovophis okinavensis* and *Trimeresurus gracilis* , as well as a closer relationship between the genera *Gloydius*, *Ovophis*, and *Trimeresurus* and the New World pitvipers. The placement of the Southeast Asian *O. monticola* and the enigmatic central‐eastern Himalayan genus *Himalayophis* was only weakly supported.

Within *Gloydius*, two main clades were recovered: a clade comprising warm‐ to cold‐temperate taxa from regions north and east of the Tibetan Plateau, spanning Central Asia, northern, eastern, and central China, the Caucasus, Korea, and Kazakhstan (Asian clade), and a high montane clade from the HHK clade. The latter included two monophyletic subclades. The first was a warm‐temperate subclade from the Hazara range—part of the Hazara‐Kashmir Syntaxis at the northwestern margin of the Himalayan orogen—and the Hindu Kush, represented by *G. hazarensis* and *G. hindukushensis*. The second comprised temperate to cold‐temperate Himalayan taxa: *G. chambensis* from the westernmost Lesser Himalaya near the Sutlej River, which was sister to a temperate to cold temperate lineage from the western and central parts of the Greater Himalaya formed by *G. himalayanus* and *G. nepalensis* .

Of note, several sequence data and the associated *Gloydius* taxa were inconsistently reported, either in GenBank or in publications, for example, the *G. rubromaculatus* holotype IOZ002317 was assigned to *G. angusticeps* (Shi et al. 2021), *G. rubromaculatus* voucher SAFS2019001 (GenBank accession numbers MW690593 and NC_064056) may represent *G. qinlingensis* (Figure 2), *G. intermedius* voucher GP188 (GenBank accession numbers JQ687469, JQ687488, JQ687507, KP403594) probably represents *G. cognatus* (Figure 2). Moreover, *Gloydius* sp. isolate CD2 and GR1 (accession numbers OK184551, OK210582, OK239647, OK239652) has been described as *G. swild* (holotype IVPPOV2725) but sequences of it were later assigned to the recently described *G. variegatus* (voucher CD2; accession numbers OK184550, OK210581, OK239647, OK239652) (Ren et al. 2025).

The stem age of *Gloydius* was estimated at *ca*. 18.6 Ma (95% HPD: 15.6–21.7 Ma), with the two main clades diverging approximately 14.5 Ma (95% HPD: 11.9–17.4 Ma) (Figure 2). Diversification within the HHK clade began during the Mid‐ to Late Miocene (11.7 Ma, 95% HPD: 8.9–14.8 Ma) and continued within its subclades until the early Pleistocene. The Central/East Asian clade began to diversify somewhat later, around the Late Miocene (9.8 Ma, 95% HPD: 7.8–12.1 Ma).

The split between the warm‐temperate subclade from the Hazara and Hindu Kush and the Himalayan subclade occurred around 11.7 Ma (95% HPD: 8.9–14.8 Ma). Subsequently, both subclades underwent nearly synchronous divergences at approximately 9.3 Ma (95% HPD: 6.3–12.7 Ma and 6.4–12.1 Ma), giving rise to *G. hindukushensis* (Hindu Kush), *G. hazarensis* (Hazara region), *G. chambensis* (Chamba valley of the Western Lesser Himalaya), *G. himalayanus* (Western Greater Himalaya), and *G. nepalensis* (Central Greater Himalaya). The crown age of the Greater Himalayan subclade comprising *G. himalayanus* and *G. nepalensis* was estimated at *ca*. 6.9 Ma (95% HPD: 4.6–9.4 Ma).

### 3.2 Niche Quantification and Species Distribution Models

**FIGURE 3:**
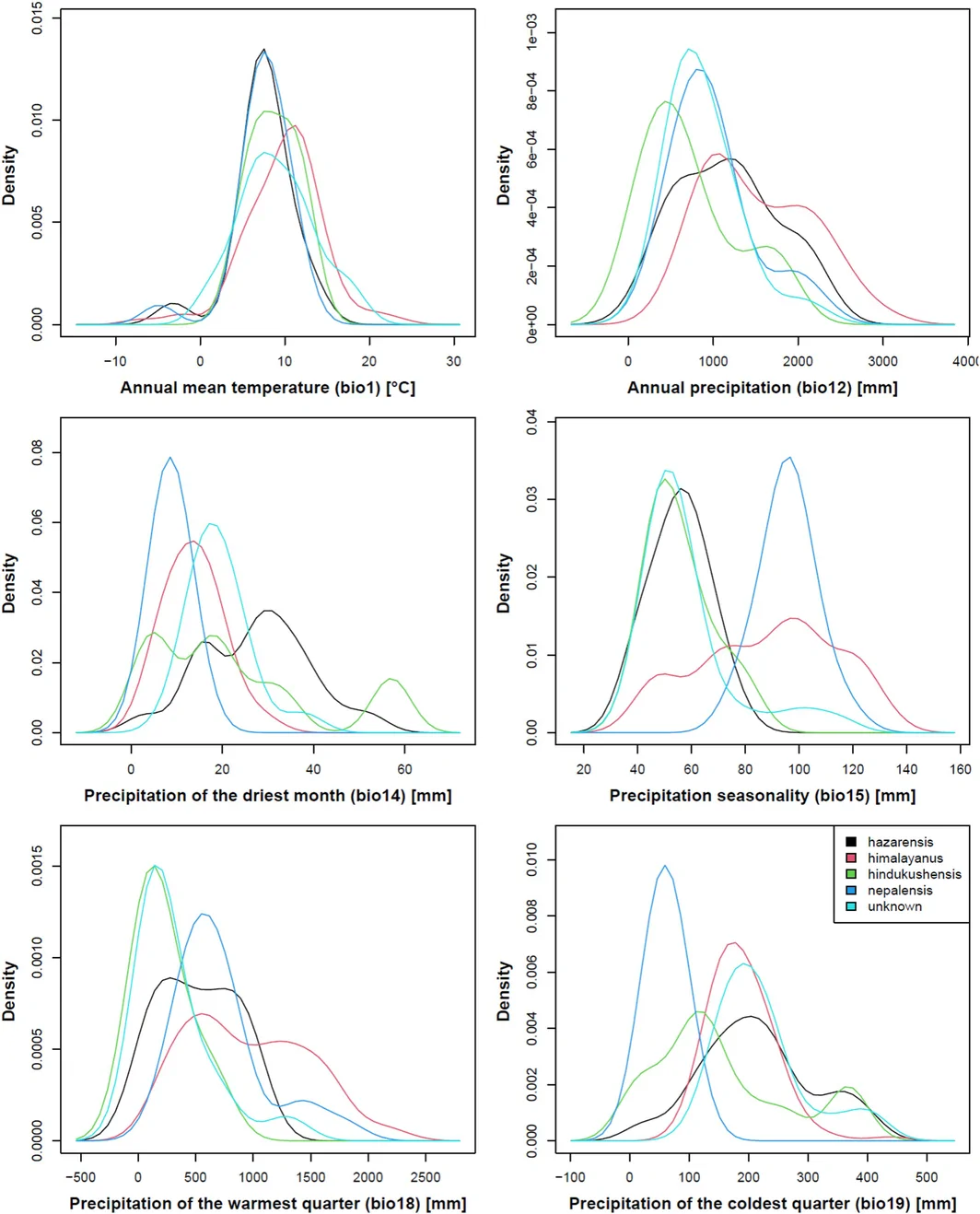
Comparison of climatic niches among *Gloydius* species based on density distributions of selected bioclimatic variables. Each curve represents the range of values occupied by different *Gloydius* species: *G. hazarensis*, *G. himalayanus* , *G. hindukushensis*, *G. nepalensis* , and unidentified populations, originally assigned to *G. himalayanus* but possibly including misidentified individuals of *G. chambensis* or *G. hazarensis*. Variables correspond to bio1 = annual mean temperature, bio12 = annual precipitation, bio14 = precipitation of driest month, bio15 = precipitation seasonality, bio18 = precipitation of warmest quarter, and bio19 = precipitation of coldest quarter.

Comparing the realized niches of all taxa revealed pronounced overlaps in niche space, which is strongest in the annual mean temperature and annual precipitation and more divergent in precipitation‐related variables (Figure 3).

Summary statistics of the SDMs are provided in Table 1. Overall, the SDMs performed well in terms of AUC and TSS scores. Across all taxa, the annual mean temperature (bio1) made the highest contribution, followed by precipitation of the warmest quarter (bio18) in *G. hazarensis* and *G. himalayanus* , and precipitation of the coldest quarter (bio19) in *G. nepalensis* . Response curves illustrating the relationship between each environmental predictor and the predicted probability of occurrence are provided in Figure S3.

**TABLE 1:** Final model settings and summary statistics of species distribution models for *Gloydius* species under study.

Species	*G. hazarensis*	*G. hindukushensis*	*G. himalayanus*	*G. nepalensis*
Feature classes	LQ	LQ	LQ	LH
Regularization	1	1.2	0.6	0.6
TSS	0.71	0.64	0.65	0.78
AUCtest	0.85	0.75	0.85	0.90
AUCtrain	0.89	0.81	0.88	0.85

Variable	Percent contribution			
bio1	58.5	83.1	70.4	47.5
bio12	1.8	0.4	2.2	6.1
bio14	0.6	8.9	3.9	2.8
bio15	2.3	2.2	3.4	7.7
bio18	34.0	4.2	13.9	7.0
bio19	2.8	1.3	6.2	28.8

**FIGURE 4:**
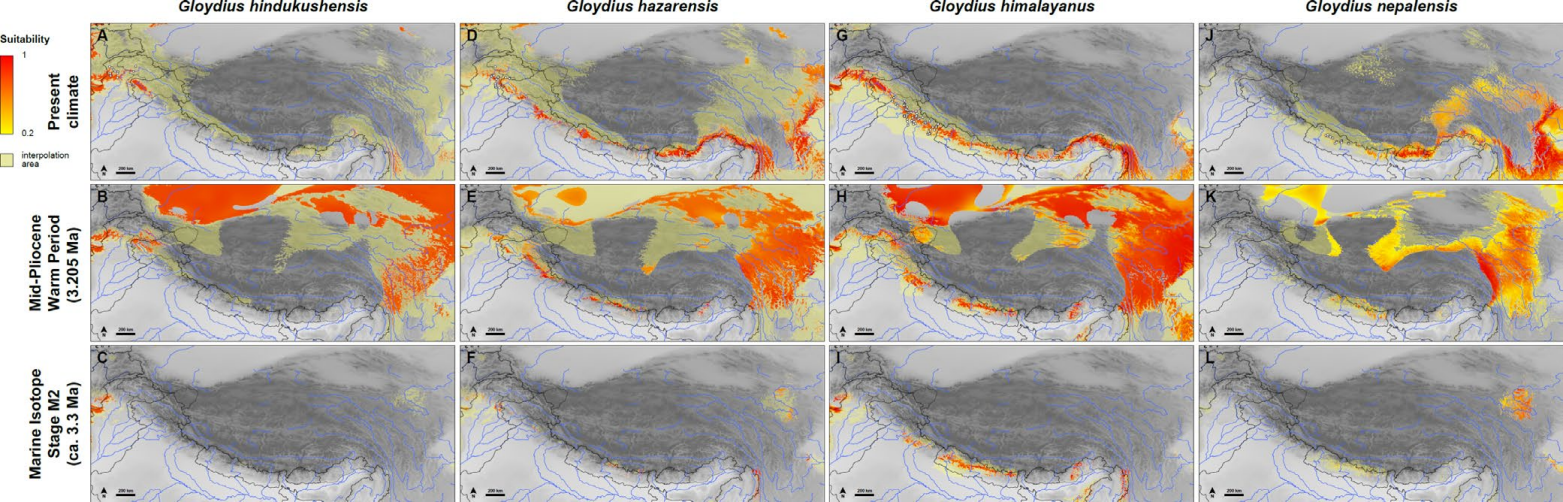
Species distribution models for four Himalayan *Gloydius* taxa under three climatic scenarios: Present, Mid‐Pliocene Warm Period (3.205 Ma), and Marine Isotope Stage M2 (*ca.* 3.3 Ma). (A–C) *G. hindukushensis*, (D–F) *G. hazarensis*, (G–I) *G. himalayanus* , and (J–L) *G. nepalensis* , shown in order as present, mPWP, and M2.

The species distribution models for four *Gloydius* taxa under different climatic scenarios revealed predicted shifts in suitable habitat across the last 3 million years, highlighting a strong potential sensitivity to paleoclimatic fluctuations (Figure 4A–L). Under present climatic conditions, *G. hindukushensis* was predicted to have a highly restricted suitable range, limited to higher‐elevation valleys in the eastern Hindu Kush (Figure 4A), which is consistent with the species' known geographic distribution (Jablonski et al. 2026) (Figure 1).

The species *G. hazarensis* was predicted to occupy mainly the southwestern margin of the Himalaya, broadly consistent with its known distribution in northern Pakistan (Figure 4D). Although the models also indicated additional suitable areas extending into the central and eastern Himalaya and the southeastern edge of the Tibetan Plateau, the species has not been recorded from these regions (Jablonski et al. 2026). Its current distribution appears geographically constrained by the Upper Indus Valley to the west, the Karakoram range to the north, and the Indus Plains to the south (Figure 1). The eastern range limit remains uncertain, particularly in the Kashmir Valley, where potential overlap with *G. chambensis* complicates range delineation and warrants further sampling and molecular analysis.

In contrast, *G. himalayanus* was predicted by the model to have a broader distribution along the Himalayan arc, extending from the westernmost ranges through the central Himalaya and into the southeastern margin of the Tibetan Plateau (Figure 4G). In reality, however, the species appears to be restricted to the Northwestern Himalaya of India, with no confirmed records outside this region (Jablonski et al. 2026) (Figure 1).

The model predicted the distribution of *G. nepalensis* to be primarily centered in the eastern Himalaya, with suitable habitats extending eastward to the southeastern margin of the Tibetan Plateau and the mountains of Indochina (Figure 4J). In contrast, the confirmed range of this taxon is limited to the central Himalaya of western and central Nepal, with verified records from the Karnali and Gandaki zones. To date, there is no credible evidence of its occurrence east of the Annapurna region (Jablonski et al. 2026) (Figure 1).

During the Mid‐Pliocene Warm Period (*ca*. 3.205 Ma), representing a globally warmer and wetter interglacial interval, the models predicted substantial expansions in suitable habitat for all four taxa (Figure 4B,E,H,K); especially *G. hazarensis*, *G. himalayanus* , and *G. hindukushensis* showed particularly extensive expansions into the northern and eastern periphery of today's Tibetan Plateau, including areas that are climatically unsuitable under current conditions. *Gloydius nepalensis* was predicted to occupy a broader range extending from the eastern Himalaya across the Hengduan Mountains and adjoining montane zones. Interestingly, despite the predicted broad‐range expansions, habitat suitability along the main Himalayan arc became more restricted according to the model. Notably, there are no known occurrences of the four species—or their close relatives—within these proposed expansion zones. Instead, their distributions are confined to the Himalaya and Hindu Kush regions.

By contrast, under the cooler and likely drier conditions of Marine Isotope Stage M2 (*ca.*3.3 Ma), all four taxa were predicted to have experienced a substantial reduction in suitable habitat (Figure 4C,F,I,L). *Gloydius* *hazarensis* and *G. hindukushensis* were modeled to persist only in small, isolated refugial areas near their present‐day range. *Gloydius himalayanus* retained a fragmented distribution across the central Himalaya, whereas *G. nepalensis* was predicted to have been restricted to a few discrete areas within the eastern and central Himalaya. The present‐day range of the latter species is centered in the western Greater Himalaya of central Nepal.

## 4 Discussion

In this study, we present the first time‐calibrated phylogeny of *Gloydius* pitvipers from the Himalaya and Hindu Kush based on mitochondrial sequence data, complemented by paleoclimatic modeling to explore the evolutionary history of the group within the framework of different concepts of Himalayan–Tibetan orogeny.

The study follows the taxonomy established in our recent integrative revision (Jablonski et al. 2026), recognizing that robust species delimitation should ideally be based on multiple independent lines of evidence rather than mitochondrial data alone (Wüster 2025).

The higher‐level relationships recovered in our topology are broadly consistent with previous studies, which have suggested a sister‐group relationship between *Gloydius* and the East Asian pitvipers *Ovophis okinavensis* and *Trimeresurus gracilis* (Malhotra and Thorpe 2004; Wüster et al. 2008; Pyron et al. 2013; Alencar et al. 2016; Pontes‐Nogueira et al. 2025). These studies, however, disagree regarding the exact placement of the genera *Protobothrops*, *Trimeresurus* sensu lato, and *Ovophis* within the subfamily Crotalinae. Most of these studies, including the present work, support a sister relationship of the New World pitvipers and the clade containing *Gloydius*. A more recent multilocus phylogenetic study based on nearly 100 molecular markers provided strong support for a sister relationship between *Ovophis* and *Gloydius*, which together form the sister clade to the green pitvipers (*Trimeresurus* sensu lato) and *Protobothrops* (Li et al. 2020). It should be noted, however, that this multilocus dataset did not include *O. okinavensis* or *T. gracilis* . A sister relationship of *Gloydius* and *Ovophis* (and a different placement of *O. okinavensis* and *T. gracilis* ) had been suggested previously, albeit with weak support (Zaher et al. 2019). Despite differences among individual studies, a clear pattern emerges regarding the early biogeography of the group: the closest extant relatives of *Gloydius* are species distributed across South, East, and Southeast Asia. Consistently, recent ancestral range estimations suggest that *Gloydius* most likely originated in Southeast Asia or the Indo‐Malayan region and that the family Viperidae as a whole has an Asian origin (Pontes‐Nogueira et al. 2025).

According to our results, *Gloydius* evolved in the early Miocene (*ca.* 18.6 Ma), with major lineage divergences between the HHK and Asian clade occurring during the Mid‐ to Late Miocene (17.4–11.9 Ma). The HHK clade began diversifying around 11.7 Ma, with subsequent splits among the five focal species dated to between *ca.* 11.7 and 6.9 Ma. These divergence ages align closely with those observed in other taxa from the Himalaya and its western adjoining mountains, ranging from herpetofauna—such as lazy toads of the genus *Scutiger* (Hofmann, Podsiadlowski, et al. 2024), spiny frogs of the genera *Chrysopaa* and *Nanorana* (Hofmann, Rödder, et al. 2024), and Himalayan lineages of the gekkonid genus *Cyrtodactylus* (Agarwal et al. 2014)—to distantly related organisms, including ground beetles (Schmidt et al. 2012, 2023), indicating comparable temporal frameworks and potentially shared biogeographic histories across multiple taxa.

These temporal patterns, consistent across multiple Himalayan taxa, raise intriguing questions about the influence of past orogenic processes and climatic fluctuations on lineage distributions. Our paleoclimatic projections over the past 3 million years illustrate the potentially highly dynamic ranges of the four *Gloydius* taxa in response to climate oscillations, highlighting their pronounced sensitivity to paleoclimate changes. This may suggest that ancestral lineages likely tracked suitable environmental niches over time, although their dispersal was constrained by the region's complex terrain within the tectonically active and topographically dynamic landscape of the HTO. Notably, because suitable paleoclimatic layers are unavailable for the Miocene, these projections are not intended as direct reconstructions of the environmental conditions associated with the inferred Miocene dispersal events, but rather as complementary evidence of historical potential range dynamics under the oldest available paleoclimatic scenarios.

Our results offer a potential temporal and spatial basis for interpreting diversification patterns in relation to geological and climatic processes in the region. In the following, we discuss alternative hypotheses for the phylogeography of *Gloydius* and evaluate their plausibility considering current scientific perspectives on the timing and mechanisms underlying the formation of the HTO.

### 4.1 Was the Tibetan Plateau Already Alpine During the Early to Mid‐Miocene, Forcing Dispersal Either East‐ or Westward Along the Himalaya?

Decades of geoscientific research across the HTO have produced a wide range of hypotheses regarding its topographic evolution, differing substantially in the timing, magnitude, and sequence of surface uplift across the orogen (reviewed in Spicer et al. 2021a). Stable isotopic paleoaltimetry studies frequently suggest that large parts of the Tibetan Plateau attained elevations close to modern values as early as the Paleogene (e.g., Rowley and Currie 2006; Quade et al. 2011; Ding et al. 2014; Xu et al. 2019), supporting models of an early high‐elevated plateau (Murphy et al. 1997; Tapponnier et al. 2001; Mulch and Chamberlain 2006; Kapp et al. 2007; Wang et al. 2008, 2014; Fang et al. 2020; Spicer et al. 2021a) and the evolution of alpine biota as early as the Oligocene (Favre et al. 2016; Ding et al. 2020). If an alpine plateau existed during this period, the ancestral lineages of today's temperate Himalayan *Gloydius* would likely have been forced to avoid these high‐elevation environments, entering the Himalaya via alternative dispersal corridors. A long‐standing hypothesis proposes that such dispersal occurred in an east–west or west–east direction parallel to the Himalaya (Martens 2015; Xu et al. 2021).

However, the phylogenetic topology within *Gloydius* reveals patterns that are unusual compared to other reptile species groups of the HTO and difficult to reconcile with either proposed geological scenarios. The closest extant relatives of Himalayan *Gloydius* occur in East Asia, east of the modern Tibetan Plateau, whereas the earliest‐diverging lineages of the genus are found in the far western Himalaya and adjacent Hindu Kush. At the time of the earliest divergence within the Himalayan clade (about 11.7 Ma), temperate conditions must have prevailed along the western edge of Paleo‐Tibet, providing suitable habitats for ancestral *Gloydius* lineages. This inference is broadly consistent with geological and paleontological evidence of a significantly uplifted Miocene Tibetan Plateau (Spicer et al. 2021a; Lai et al. 2025), irrespective of whether this plateau had already attained alpine elevations by that time.

From a paleogeographic and paleoecological perspective, the timing of divergence between the Himalayan and East Asian *Gloydius* clade (*ca.* 14.5 Ma; 11.9–17.4 Ma) and the first cladogenetic event within the Himalayan *Gloydius* (HHK clade; *ca.* 11.7 Ma; 8.9–14.8 Ma) are particularly noteworthy. During this interval, the ancestor of the HHK clade must have colonized the western margin of Paleo‐Tibet. If the plateau had already attained alpine elevations by that time, dispersal of ancestral *Gloydius* may have been possible only along the southern slope of the HTO. However, *Gloydius* occurs today exclusively in the western and, with a single terminal(!) clade, in the central Himalaya but is entirely absent from the eastern Himalaya. For the latter region to have once been inhabited, ancestral populations would have had to be extirpated from the entire central and eastern Himalayan arc—an unlikely scenario given the presence of multiple *Gloydius* species along the eastern margin of the Tibetan Plateau and the complete absence of the genus from the eastern Himalaya. This interpretation is further supported by species distribution models, which predict potentially suitable habitats for *G. hazarensis*, *G. himalayanus* , and *G. nepalensis* in the eastern Himalaya that appear to remain unoccupied. Collectively, these findings suggest that the striking absence of *Gloydius* from the eastern Himalaya and the lack of early‐diverging lineages in the central Himalaya cannot be explained by a dispersal route confined to the Himalayan arc.

A dispersal route parallel to the Himalayan arc would have necessitated that ancestral *Gloydius* traversed the north–south oriented mountain ranges and deeply incised transverse valleys that have characterized the region since its initial uplift. Rivers and mountains, however, are well established as effective barriers to snake dispersal, including pitvipers (Jablonski et al. 2026; Lee et al. 2024; Moradi et al. 2024). Although crossing some deeply incised transverse valleys may have been possible—facilitated by the geomorphodynamics of the Himalaya, such as landslides and valley infilling (Fort 1996, 2000)—continuous east–west dispersal along the entire Himalayan arc would have constituted an enormous, and likely insurmountable, barrier for *Gloydius*. Moreover, such a scenario should have left phylogenetic signatures, at minimum in the form of old, relic lineages in parts of the eastern and central Himalaya, which are entirely absent. Thus, accounting for the ancestral origins of the Himalayan and Hindu Kush species—and the routes by which they arrived—requires consideration of alternative scenarios for long‐distance dispersal in *Gloydius*.

### 4.2 Warm‐Temperate Corridors Across Paleo‐Tibet as Potential Dispersal Pathways Into the Rising Himalaya?

The estimated crown age of *Gloydius* (*ca.* 14.5 Ma) in our dated phylogeny marks the earliest possible existence of a shared ancestral range for the present‐day Himalayan clade and its Asian sister group. Phylogenetic relationships and current distributions strongly suggest that their common ancestor inhabited tropical to warm‐temperate environments in East and Southeast Asia, consistent with earlier reconstructions (Pontes‐Nogueira et al. 2025). This raises two central questions: (i) if east–west dispersal parallel to the Himalaya is implausible, by which routes did ancestral *Gloydius* colonize the western Himalaya and Hindu Kush following divergence from their Asian relatives east of the modern Tibetan Plateau, and (ii) what ecological and evolutionary processes occurred during the interval between this divergence and the first split within the HHK clade?

Answering these questions requires integration of recent paleontological evidence with reconstructions of Tibet's paleoenvironmental history. Contrary to models proposing a highly elevated Paleogene Tibetan Plateau, the Cenozoic fossil record of the HTO (compiled in Spicer et al. 2021b; Schmidt et al. 2023; Lai et al. 2025) indicates that large parts of Tibet remained at comparatively low elevations and supported subtropical to temperate biomes at least until the Early Miocene (reviewed in Ai et al. 2019; Deng et al. 2019). Importantly, no fossil evidence currently substantiates cold‐temperate or alpine habitats in central or southern Paleo‐Tibet until the end of the Paleogene. This absence casts doubt on early high‐elevation scenarios and, by extension, undermines models that constrain dispersal exclusively to the Himalayan arc.

We therefore propose that, beginning in the early to mid‐Miocene, the emergence of warm‐temperate habitats across Paleo‐Tibet created ecologically suitable corridors that enabled westward dispersal of ancestral *Gloydius*. These corridors may have facilitated their separation from tropical–subtropical congeners, ultimately giving rise to the distinct Himalayan clade. A comparable paleogeographic scenario has previously been proposed for the evolutionary history of Himalayan amphibians of the genera *Chrysopaa*, *Nanorana*, and *Scutiger* (Hofmann, Podsiadlowski, et al. 2024; Hofmann, Rödder, et al. 2024; see Schmidt et al. 2023 for details of the scenario). Fossil evidence supports this interpretation: the Namling flora documents temperate forests in the central Gangdese Shan during the Mid‐Miocene (*ca.* 15 Ma; Zhou et al. 2007), while subtropical to warm‐temperate fossil floras have been found in the Lunpola and Qiabulin basins in central‐southern Tibet, and dated to the Oligocene‐Miocene boundary/late early Miocene (e.g., Sun et al. 2014; Ding et al. 2017; Wu et al. 2017). Moreover, geochemical data from the Qaidam Basin (Sun, Liang, et al. 2023) indicate that uplift to present elevations (*ca.* 3000 m) occurred at the earliest in the Mid‐Miocene, reinforcing the view that east–west warm‐temperate “niche corridors” were available to Asian biota (Spicer et al. 2021a; Schmidt et al. 2023). Recent geoscientific work even emphasizes a warm‐humid climate in central Tibet persisting until the Mid‐Miocene, with peak humidity around 15–14 Ma (Sun, Li, et al. 2023).

Nevertheless, knowledge of past environments and elevations across different tectonic domains of Tibet remains fragmentary and controversial (compiled in Lai et al. 2025). For example, reconstructions for central Tibet diverge widely: some infer elevations exceeding 4000–5000 m already in the late Eocene/early Oligocene (Rowley and Currie 2006; Xiong et al. 2022), while others suggest < 2300 m in the latest Oligocene (Wu et al. 2017; Su et al. 2019), *ca.* 3000 m in the early Miocene (Deng et al. 2012; Sun et al. 2014), or < 2500 m and > 4500 m during the middle Miocene (Spicer et al. 2003; Sun, Li, et al. 2023). Such discrepancies stem from methodological differences as well as from uncertainties in paleoelevational and paleogeographic reconstructions. Competing models—such as PALEOMAP (Scotese and Wright 2018), GPlates (Müller et al. 2016; Zahirovic et al. 2022), or Getech's Globe/Neftex reconstructions—adopt contrasting assumptions on the timing and style of uplift (and the resulting topography), ranging from an “early high plateau” to more gradual, mosaic patterns of elevation. Consequently, no single geoscientific framework can yet be regarded as definitive. This underlines the value of dated phylogenetic patterns, such as those in *Gloydius*, which provide an independent biological archive that can contribute to disentangle the complex paleoenvironmental history of the HTO.

**FIGURE 5:**
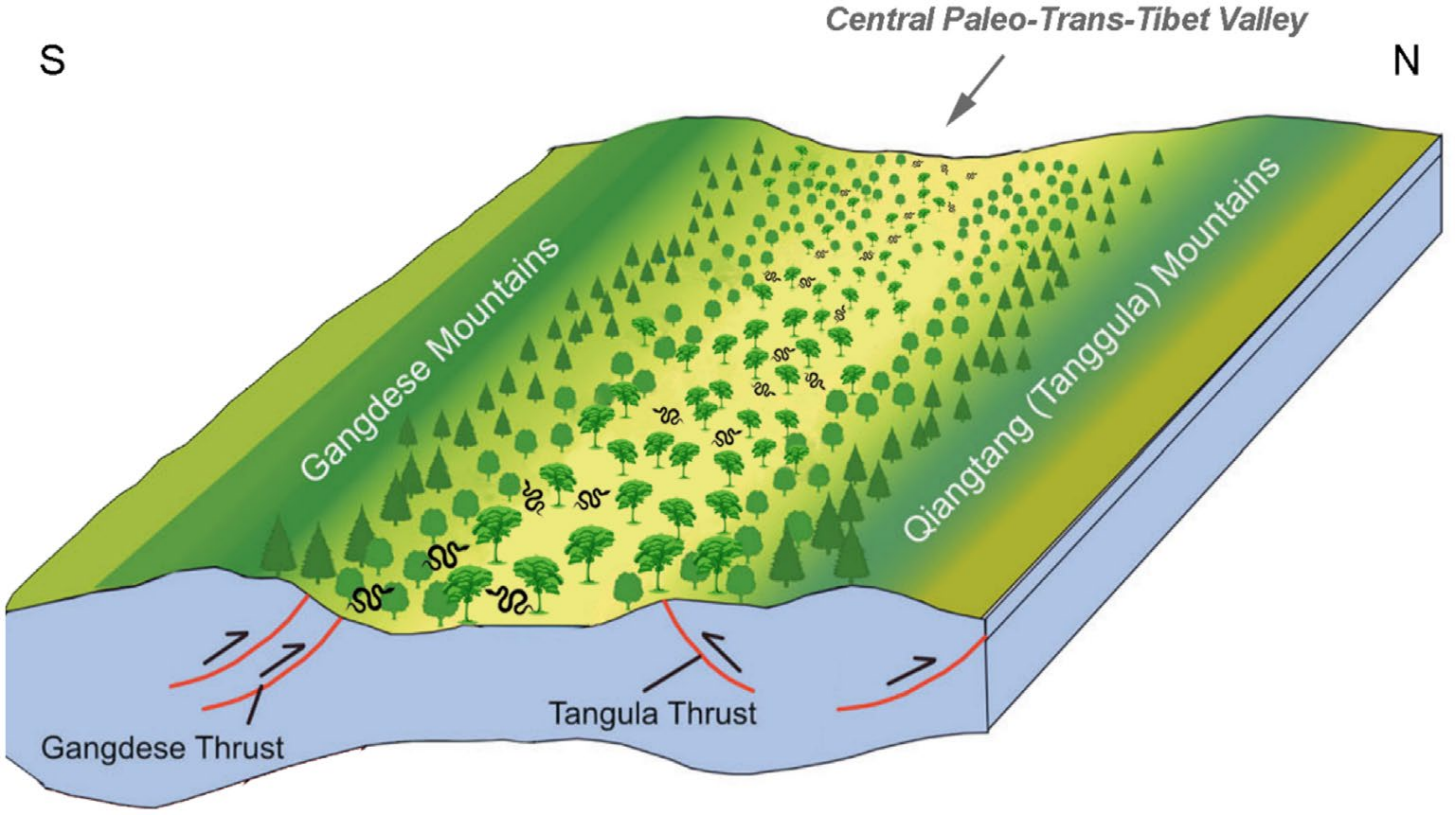
Schematic illustration of the low‐elevation Central Paleo‐Trans‐Tibet valley. We assume the existence of such a subtropical to warm‐temperate corridor for the early to middle Miocene (figure modified from Spicer et al. 2021a).

Assuming an east–west trans‐Tibetan movement of subtropical to warm‐temperate ancestral *Gloydius* lineages during the Miocene, their presence across paleo‐Tibet may have been facilitated by an east–west‐oriented low‐elevation zone in central paleo‐Tibet characterized by a subtropical to temperate climate. The most recent geoscientific model, supported by fossil evidence, indicates the existence of such suitable environments between the east–west‐trending Gangdese and Tanggula Mountains, the latter forming the present‐day Qiangtang Terrane (Spicer et al. 2021a). We refer to this corridor hereafter as the Central Paleo‐Trans‐Tibet Valley (Figure 5). It should be noted that nearly two decades ago, subtropical primary Tertiary ranges along the Transhimalaya had already been proposed for ground beetles endemic to the Himalaya (Schmidt 2006)—a model later considered most plausible for explaining the historical biogeography of Himalayan‐endemic amphibians (Hofmann et al. 2017, 2019).

The Gangdese Mountains likely represented a southern barrier for species inhabiting this valley—provided these mountains had already attained considerable elevation at that time. It is plausible that ancestral *Gloydius* lineages occupied this valley extensively during the Middle Miocene and reached as far as the western slopes of the Gangdese Mountains. During the Late Miocene, the Central Paleo‐Trans‐Tibet Valley was further elevated and, based on the paleontological record, characterized by high montane forests adapted to colder temperatures (“alpine forest” sensu Lai et al. 2025). This environmental change may have displaced *Gloydius* populations toward the opposite margins of the Himalayan–Tibetan Orogen (HTO), leading to the evolution of the western clade (the HHK clade) now separated from its eastern relatives.

Regardless of the exact elevation of the surrounding terrain, temperate habitats must have remained widespread along western slopes of paleo‐Tibet and the rising Himalaya—such as along the catchment areas of the paleo‐Indus River—allowing these snakes to survive and expand their geographic ranges along drainage systems that formed during the further uplift in this part of the HTO. The Indus River, originating in western Tibet and the Karakoram, predates the main phase of Himalayan uplift and is among the oldest documented rivers in the HTO (Clift et al. 2001; Inam et al. 2007). These pre‐existing and newly formed drainage systems likely facilitated the dispersal of ancestral *Gloydius* along the intramontane Indus–Yarlung Suture Zone, which extended from northwest to southeast between the Gangdese Mountains and the rising Himalaya. This corridor would have enabled southward and eastward range expansion of *Gloydius*, while its western continuation probably corresponded to the present‐day Indus River valley. By following this moderately elevated longitudinal valley of the Indus–Yarlung Suture Zone, *Gloydius* may have accessed epigenetic Himalayan transverse valleys, such as the Sutlej, Karnali, and Thakkhola, subsequently colonizing the rising Himalaya.

The absence of *Gloydius* in the eastern Himalaya, together with the genus' current distributional limit in the Himalaya of central Nepal, may indicate that the highest‐elevation section of this ancient corridor acted as a barrier to ancestral *Gloydius*, although this remains speculative. Interestingly, other Tertiary West‐Tibetan faunal elements of the Himalaya (i.e., faunal groups primarily distributed in the western Himalaya with a Tertiary origin in West Tibet) also reach their eastern range limits in the Nepal Himalaya, for example, ground beetles of the genera *Calathus*, *Carabus*, and *Pristosia* (Schmidt 1999; Schmidt and Hartmann 2009; Schmidt et al. 2023)—a pattern not readily explained by climatic factors. Consistently, our species distribution models reveal climatically suitable habitats throughout the eastern Himalaya, yet *Gloydius* remains entirely absent, with no records from this region.

Divergence among the ancestral lineages then occurred after their arrival in the rising Himalaya, driven by continuous uplift and the resulting increase in topographic barriers, leading to allopatric separation. According to our dated phylogenetic tree, this process took place from the mid‐ to late Miocene (*ca.*11.7–6.9 Ma), a timeframe that aligns with most geoscientific models of Himalayan uplift (Molnar et al. 1993; Tapponnier et al. 2001; Song et al. 2024). The ongoing uplift of the Tibetan Plateau ultimately resulted in the loss of ancestral *Gloydius*' primary ranges across paleo‐Tibet driven by the associated cooling and aridification, a pattern similarly observed in other endemic Himalayan taxa (Schmidt et al. 2016, 2023; Hofmann, Podsiadlowski, et al. 2024; Hofmann, Rödder, et al. 2024).

## 5 Conclusion

This study provides a framework for the timing and spatial patterns of Himalayan *Gloydius* evolution, integrating a time‐calibrated phylogeny with paleoclimatic and geological evidence. Our results suggest that ancestral lineages may have expanded across paleo‐Tibet during the Miocene via subtropical to warm‐temperate, low‐elevation corridors. The Central Paleo‐Trans‐Tibet Valley, situated between the Gangdese and Tanggula Mountains, may have served as the primary route facilitating westward movement from East and Southeast Asia, where the closest extant relatives of Himalayan *Gloydius* occur.

As the plateau continued to uplift during the Late Miocene, high montane environments and emerging topographic barriers may have fragmented populations. A secondary corridor along the intramontane Indus–Yarlung Suture Zone may have guided lineages into epigenetic transverse valleys, which acted as the primary entrance into the rising Himalaya. Through these valleys, *Gloydius* may have colonized the western and central Himalayan ranges, while the eastern Himalaya remained unoccupied, probably due to persistent geographic barriers, despite climatically suitable habitats. Once established within the Himalayan ranges, ancestral *Gloydius* may have undergone allopatric divergence in response to increasing topographic barriers during the ongoing Himalayan uplift.

By providing a plausible scenario for the paradoxical phylogenetic topology of *Gloydius*, this study offers new insights into the origin, historical biogeography, and diversification of these taxa. Importantly, it presents a biogeographic model explaining the disjunct trans‐Tibet distributions of low‐dispersal organisms, suggesting specific paleoenvironmental corridors across paleo‐Tibet that may have guided their movement and persistence, offering a biotic perspective on the Miocene tectonic and environmental evolution of the Himalayan–Tibetan Orogen.

## Author Contributions

**Sylvia Hofmann:** conceptualization (lead), formal analysis (equal), funding acquisition (lead), investigation (equal), methodology (equal), project administration (lead), resources (equal), visualization (equal), writing – original draft (lead), writing – review and editing (equal). **Elán Pérez García:** formal analysis (equal), investigation (equal), methodology (equal), writing – review and editing (equal). **Morris Flecks:** visualization (equal), writing – review and editing (equal). **Daniel Jablonski:** investigation (equal), funding acquisition (equal), resources (equal), writing – review and editing (equal). **Chitra B. Baniya:** investigation (equal), resources (equal), writing – review and editing (equal). **Dennis Rödder:** conceptualization (equal), data curation (equal), formal analysis (equal), investigation (equal), methodology (equal), writing – review and editing (equal). **Joachim Schmidt:** conceptualization (equal), investigation (equal), writing – review and editing (equal).

## Conflicts of Interest

The authors declare no conflicts of interest.

## Supporting information


**Figure S1:** Maximum‐likelihood tree generated with IQ‐TREE 2 from 2693 bp of concatenated mitochondrial sequences.
**Figure S2:** Time‐calibrated phylogeny of *Gloydius* with selected outgroups [high‐resolution version of Figure 2].
**Figure S3:** Maxent response curves for the study species.
**Table S1:** Geographic records used for the map (Figure 1) and species distribution modeling. All coordinates are provided in decimal degrees (WGS84 datum).
**Table S2:** List of *Gloydius* (G.) species and outgroups (outgr.) included in this study.

## Data Availability

All sequence data are available in GenBank. Species occurrence records are provided in the Supporting Information. Analytical scripts are available in the appendix of Noori et al. (2025).
