# Supplementary material for: Tracing Ancient Migration Routes: Did Gloydius Pitvipers Disperse Across Paleo‐Tibet Through Temperate and Subtropical Corridors?
Source: Ecol Evol. 2026 Sep 14;16(9):e74238. doi: 10.1002/ece3.74238 (PMC13573089; doi:10.1002/ece3.74238)
Supplement: Supplementary file 1 [file ECE3-16-e74238-s001.zip › 3_#Suppl_FigS2_450dpi.pdf]

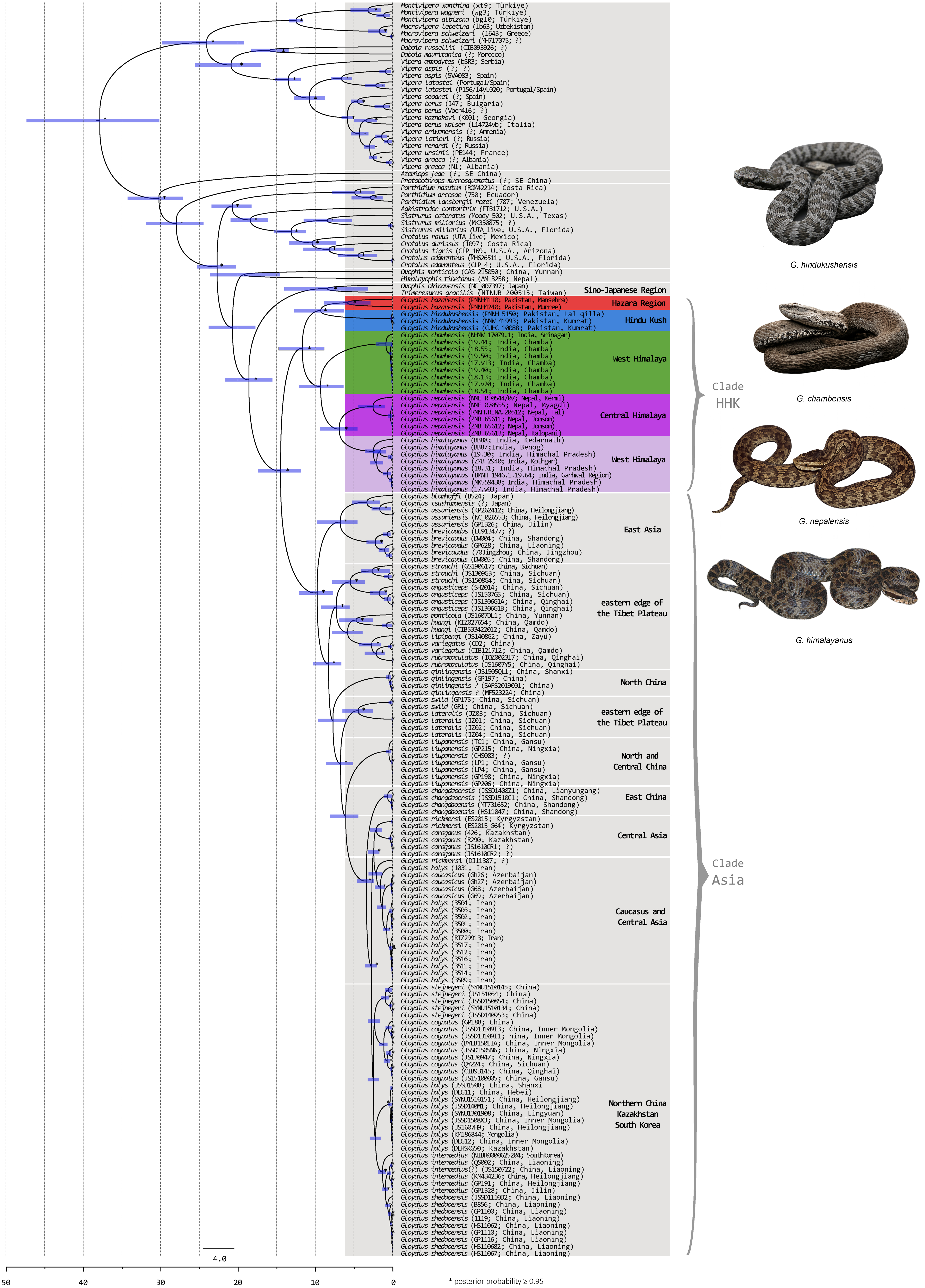

Montivipera xanthina (xt9; Türkiye)
Montivipera wagneri (wg3; Türkiye)
Montivipera albizona (bg10; Türkiye)
Macrovipera lebetina (lb63; Uzbekistan)
Macrovipera schweizeri (1643; Greece)
Macrovipera schweizeri (MH717075; ?)
Daboia russellii (CIB093926; ?)
Daboia mauritanica (?; Morocco)
Vipera ammodytes (bSR3; Serbia)
Vipera aspis (?; ?)
Vipera aspis (SVA083; Spain)
Vipera latastei (Portugal/Spain)
Vipera latastei (P156/14VL020; Portugal/Spain)
Vipera seoanei (?; Spain)
Vipera berus (J47; Bulgaria)
Vipera berus (Vber416; ?)
Vipera kaznakovi (K001; Georgia)
Vipera berus walser (L14724vb; Italia)
Vipera eriwanensis (?; Armenia)
Vipera lotievi (?; Russia)
Vipera renardi (?; Russia)
Vipera ursinii (PE144; France)
Vipera graeca (?; Albania)
Vipera graeca (N1; Albania)
Azemiops feae (?; SE China)
Protobothrops mucrosquamatus (?; SE China)
Porthidium nasutum (ROM42214; Costa Rica)
Porthidium arcosae (750; Ecuador)
Porthidium lansbergii rozei (787; Venezuela)
Agkistrodon contortrix (FTB1712; U.S.A.)
Sistrurus catenatus (Moody 502; U.S.A., Texas)
Sistrurus miliarius (MK330875; ?)
Sistrurus miliarius (UTA live; U.S.A., Florida)
Crotalus ravus (UTA live; Mexico)
Crotalus durissus (1097; Costa Rica)
Crotalus tigris (CLP 169; U.S.A., Arizona)
Crotalus adamanteus (MH626511; U.S.A., Florida)
Crotalus adamanteus (CLP 4; U.S.A., Florida)
Ovophis monticola (CAS 215050; China, Yunnan)
Himalayophis tibetanus (AM B258; Nepal)
Ovophis okinavensis (NC 007397; Japan)
Trimeresurus gracilis (NTNUB 200515; Taiwan)
Sino-Japanese Region
Gloydius hazarensis (PMNH4110; Pakistan, Mansehra)
Gloydius hazarensis (PMNH4240; Pakistan, Murree)
Hazara Region
Gloydius hindukushensis (PMNH 5150; Pakistan, Lal qilla)
Gloydius hindukushensis (NMW 41993; Pakistan, Kumrat)
Gloydius hindukushensis (CUHC 10888; Pakistan, Kumrat)
Hindu Kush
Gloydius chambensis (NHMW 17079.1; India, Srinagar)
Gloydius chambensis (19.44; India, Chamba)
Gloydius chambensis (18.55; India, Chamba)
Gloydius chambensis (19.50; India, Chamba)
Gloydius chambensis (17.v13; India, Chamba)
Gloydius chambensis (19.40; India, Chamba)
Gloydius chambensis (18.13; India, Chamba)
Gloydius chambensis (17.v20; India, Chamba)
Gloydius chambensis (18.54; India, Chamba)
West Himalaya
Gloydius nepalensis (NME R 0544/07; Nepal, Kermi)
Gloydius nepalensis (NME 070555; Nepal, Myagdi)
Gloydius nepalensis (RMNH.RENA.20512; Nepal, Tal)
Gloydius nepalensis (ZMB 65611; Nepal, Jomsom)
Gloydius nepalensis (ZMB 65612; Nepal, Jomsom)
Gloydius nepalensis (ZMB 65613; Nepal, Kalopani)
Central Himalaya
Gloydius himalayanus (BB88; India, Kedarnath)
Gloydius himalayanus (BB87;India, Benog)
Gloydius himalayanus (19.30; India, Himachal Pradesh)
Gloydius himalayanus (ZMB 2940; India, Kothgar)
Gloydius himalayanus (18.31; India, Himachal Pradesh)
Gloydius himalayanus (BMNH 1946.1.19.64; India, Garhwal Region)
Gloydius himalayanus (MK559438; India, Himachal Pradesh)
Gloydius himalayanus (17.v03; India, Himachal Pradesh)
West Himalaya
Gloydius blomhoffi (B524; Japan)
Gloydius tsushimaensis (?; Japan)
Gloydius ussuriensis (KP262412; China, Heilongjiang)
Gloydius ussuriensis (NC_026553; China, Heilongjiang)
Gloydius ussuriensis (GP1326; China, Jilin)
Gloydius brevicaudus (EU913477; ?)
Gloydius brevicaudus (DW004; China, Shandong)
Gloydius brevicaudus (GP628; China, Liaoning)
Gloydius brevicaudus (70Jingzhou; China, Jingzhou)
Gloydius brevicaudus (DW005; China, Shandong)
East Asia
Gloydius strauchi (GS190617; China, Sichuan)
Gloydius strauchi (JS1309G3; China, Sichuan)
Gloydius strauchi (JS1508G4; China, Sichuan)
Gloydius angusticeps (SH2014; China, Sichuan)
Gloydius angusticeps (JS1507G5; China, Sichuan)
Gloydius angusticeps (JS1306G1A; China, Qinghai)
Gloydius angusticeps (JS1306G1B; China, Qinghai)
Gloydius monticola (JS1607DL1; China, Yunnan)
Gloydius huangi (KIZ027654; China, Qamdo)
Gloydius huangi (CIB533422012; China, Qamdo)
Gloydius lipipengi (JS1408G2; China, Zayü)
Gloydius variegatus (CD2; China)
Gloydius variegatus (CIB121712; China, Qamdo)
Gloydius rubromaculatus (IOZ002317; China, Qinghai)
Gloydius rubromaculatus (JS1607Y5; China, Qinghai)
eastern edge of
the Tibet Plateau
Gloydius qinlingensis (JS1505QL1; China, Shanxi)
Gloydius qinlingensis (GP197; China)
Gloydius qinlingensis ? (SAFS2019001; China)
Gloydius qinlingensis ? (MF523224; China)
North China
Gloydius swild (GP175; China, Sichuan)
Gloydius swild (GR1; China, Sichuan)
Gloydius lateralis (JZ03; China, Sichuan)
Gloydius lateralis (JZ01; China, Sichuan)
Gloydius lateralis (JZ02; China, Sichuan)
Gloydius lateralis (JZ04; China, Sichuan)
eastern edge of
the Tibet Plateau
Gloydius liupanensis (TC1; China, Gansu)
Gloydius liupanensis (GP215; China, Ningxia)
Gloydius liupanensis (CHS083; ?)
Gloydius liupanensis (LP1; China, Gansu)
Gloydius liupanensis (LP4; China, Gansu)
Gloydius liupanensis (GP198; China, Ningxia)
Gloydius liupanensis (GP206; China, Ningxia)
North and
Central China
Gloydius changdaoensis (JSSD1408Z1; China, Lianyungang)
Gloydius changdaoensis (JSSD1510C1; China, Shandong)
Gloydius changdaoensis (MT731652; China, Shandong)
Gloydius changdaoensis (HS11047; China, Shandong)
East China
Gloydius rickmersi (ES2015; Kyrgyzstan)
Gloydius rickmersi (ES2015 G64; Kyrgyzstan)
Gloydius caraganus (426; Kazakhstan)
Gloydius caraganus (R290; Kazakhstan)
Gloydius caraganus (JS1610CR1; ?)
Gloydius caraganus (JS1610CR2; ?)
Central Asia
Gloydius rickmersi (DJ11387; ?)
Gloydius halys (1031; Iran)
Gloydius caucasicus (Gh26; Azerbaijan)
Gloydius caucasicus (Gh27; Azerbaijan)
Gloydius caucasicus (G68; Azerbaijan)
Gloydius caucasicus (G69; Azerbaijan)
Gloydius halys (3504; Iran)
Gloydius halys (3503; Iran)
Gloydius halys (3502; Iran)
Gloydius halys (3501; Iran)
Gloydius halys (3500; Iran)
Gloydius halys (RIZ29913; Iran)
Gloydius halys (3517; Iran)
Gloydius halys (3512; Iran)
Gloydius halys (3516; Iran)
Gloydius halys (3511; Iran)
Gloydius halys (3514; Iran)
Gloydius halys (3509; Iran)
Caucasus and
Central Asia
Gloydius stejnegeri (SYNU1510145; China)
Gloydius stejnegeri (JS151054; China)
Gloydius stejnegeri (JSSD1508S4; China)
Gloydius stejnegeri (SYNU1510134; China)
Gloydius stejnegeri (JSSD1409S3; China)
Gloydius cognatus (GP188; China)
Gloydius cognatus (JSSD13109I3; China, Inner Mongolia)
Gloydius cognatus (JSSD13109I1; hina, Inner Mongolia)
Gloydius cognatus (BYEB1501IA; China, Inner Mongolia)
Gloydius cognatus (JSSD1505N6; China, Ningxia)
Gloydius cognatus (JS130947; China, Ningxia)
Gloydius cognatus (QY224; China, Sichuan)
Gloydius cognatus (CIB93145; China, Qinghai)
Gloydius cognatus (JS15100005; China, Gansu)
Gloydius halys (JSSD1508; China, Shanxi)
Gloydius halys (DLG11; China, Hebei)
Gloydius halys (SYNU1510151; China, Heilongjiang)
Gloydius halys (JSSD140M1; China, Heilongjiang)
Gloydius halys (SYNU1301908; China, Lingyuan)
Gloydius halys (JSSD1508X3; China, Inner Mongolia)
Gloydius halys (JS1607H9; China, Heilongjiang)
Gloydius halys (KM186844; Mongolia)
Gloydius halys (DLG12; China, Inner Mongolia)
Gloydius halys (DLHSKG50; Kazakhstan)
Gloydius intermedius (NIBR000625204; SouthKorea)
Gloydius intermedius (QS002; China, Liaoning)
Gloydius intermedius(?) (JS150722; China, Liaoning)
Gloydius intermedius (KM434236; China, Heilongjiang)
Gloydius intermedius (GP191; China, Heilongjiang)
Gloydius intermedius (GP1328; China, Jilin)
Gloydius shedaoensis (JSSD1110D2; China, Liaoning)
Gloydius shedaoensis (B856; China, Liaoning)
Gloydius shedaoensis (GP1100; China, Liaoning)
Gloydius shedaoensis (1119; China, Liaoning)
Gloydius shedaoensis (HS11062; China, Liaoning)
Gloydius shedaoensis (GP1110; China, Liaoning)
Gloydius shedaoensis (GP1116; China, Liaoning)
Gloydius shedaoensis (HS110682; China, Liaoning)
Gloydius shedaoensis (HS11067; China, Liaoning)
Northern China
Kazakhstan
South Korea
G. hindukushensis
G. chambensis
G. nepalensis
G. himalayanus
Clade
HHK
Clade
Asia
4.0
50
40
30
20
10
0
* posterior probability ≥ 0.95
