# Supplementary material for: Tracing Ancient Migration Routes: Did Gloydius Pitvipers Disperse Across Paleo‐Tibet Through Temperate and Subtropical Corridors?
Source: Ecol Evol. 2026 Sep 14;16(9):e74238. doi: 10.1002/ece3.74238 (PMC13573089; doi:10.1002/ece3.74238)

Supporting Information for

Tracing ancient migration routes: Did pitvipers of the genus *Gloydius* disperse across Paleo-Tibet through temperate and subtropical corridors?

Hofmann S, García E, Flecks M, Jablonski D, Baniya CB, Rödder D, Schmidt J

Table of content

**Supplementary Table S1**: Geographic records used for the map (Fig. 1) and species distribution modelling. All coordinates are provided in decimal degrees (WGS84 datum).	p.1
**Supplementary Table S2**: List of *Gloydius* (*G.*) species and outgroups (outgr.) included in this study.	p.5
**Supplementary Figure S1**: Maximum-likelihood tree generated with IQ-TREE 2 from 2,693 bp of concatenated mitochondrial sequences.	p.9
**Supplementary Figure S2**: Time-calibrated phylogeny of *Gloydius* with selected outgroups. [high-resolution version of Fig. 2]	separate file
**Supplementary Fig. S3**: Maxent response curves for the study species.	p.10

Supplementary Table S1. Geographic records used for the map (Fig. 1) and species distribution modelling. All coordinates are provided in decimal degrees (WGS84 datum).

No.	E	N	elevation_m	species	Country	Region
1	71.8000000	35.8833330	3048	*hindukushensis*	Pakistan	Chitral District, Khyber Pakhtunkhwa Province
2	72.0255556	35.7752778	2650	*hindukushensis*	Pakistan	Lower Chitral District, Khyber Pakhtunkhawa Province
3	74.3050000	35.9169444	~1500	*hindukushensis*	Pakistan	Gilgit District, Gilgit-Baltistan Province
4	74.0986111	35.4219444	1180	*hindukushensis*	Pakistan	Diamer District, Gilgit-Baltistan Province
5	72.1958310	35.5649030	2360	*hindukushensis*	Pakistan	Upper Dir District, Khyber Pakhtunkhwa Province
6	71.8605556	35.2233333	1764–2119	*hindukushensis*	Pakistan	Upper Dir District, Khyber Pakhtunkhwa Province
7	72.3202778	35.4438889	2490–2888	*hindukushensis*	Pakistan	Upper Dir District, Khyber Pakhtunkhwa Province
8	71.8694201	35.0337557	2446	*hindukushensis*	Pakistan	Upper Dir District, Khyber Pakhtunkhwa Province
9	71.9030127	34.9898497	2700	*hindukushensis*	Pakistan	Upper Dir District, Khyber Pakhtunkhwa Province
10	72.5980556	35.3933333	2100	*hindukushensis*	Pakistan	Swat District, Khyber Pakhtunkhwa Province
11	72.5480630	34.8044310	1850	*hindukushensis*	Pakistan	Swat District, Khyber Pakhtunkhwa Province
12	72.5674250	35.2366310	1660	*hindukushensis*	Pakistan	Swat District, Khyber Pakhtunkhwa Province
13	73.0936110	34.9897222	2400–2650	*hazarensis*	Pakistan	Kolai-Palas District, Khyber Pakhtunkhwa Province
14	73.0458330	34.9958333	2400	*hazarensis*	Pakistan	Kolai-Palas District, Khyber Pakhtunkhwa Province
15	73.0058333	35.0016667	2750	*hazarensis*	Pakistan	Kolai-Palas District, Khyber Pakhtunkhwa Province
16	73.2233300	35.0219444	2500	*hazarensis*	Pakistan	Kolai-Palas District, Khyber Pakhtunkhwa Province
17	72.9908330	34.9688889	2500-2700	*hazarensis*	Pakistan	Kolai-Palas District, Khyber Pakhtunkhwa Province
18	74.3578103	34.8352514	2256	*hazarensis*	Pakistan	Neelam District, Azad Kashmir State
19	74.7463145	34.7478186	2385	*hazarensis*	Pakistan	Neelam District, Azad Kashmir State
20	74.7119444	34.7255556	~2300	*hazarensis*	Pakistan	Neelam District, Azad Kashmir State
21	73.7513880	33.8533333	~1630	*hazarensis*	Pakistan	Poonch District, Azad Kashmir State
22	73.4364900	34.7048500	2463	*hazarensis*	Pakistan	Mansehra District, Khyber Pakhtunkhwa Province
23	73.6458333	34.8986110	2400	*hazarensis*	Pakistan	Mansehra District, Khyber Pakhtunkhwa Province
24	73.6272222	34.8805556	~2200	*hazarensis*	Pakistan	Mansehra District, Khyber Pakhtunkhwa Province
25	73.4636111	34.6411111	2350	*hazarensis*	Pakistan	Mansehra District, Khyber Pakhtunkhwa Province
26	73.3811111	34.0730556	2450	*hazarensis*	Pakistan	Abbottabad District, Khyber Pakhtunkhawa Province
27	73.3500000	34.2333333	2750	*hazarensis*	Pakistan	Abbottabad District, Khyber Pakhtunkhawa Province
28	73.6128921	34.5625541	2900	*hazarensis*	Pakistan	Bagh District, Azad Kashmir State
29	73.3902778	33.9041667	2300	*hazarensis*	Pakistan	Murree District, Punjab Province
30	73.3374990	33.8763889	1800	*hazarensis*	Pakistan	Murree District, Punjab Province
31	73.4269440	33.8968070	1738	*hazarensis*	Pakistan	Rawalpindi District, Punjab Province
32	74.7711320	34.6147400	3520	?	India	Kupwara District, Jammu and Kashmir State
33	74.8333000	34.6333000	2580	?	India	Bandipora District, Jammu and Kashmir State
34	74.6994444	34.6436111	2400	?	India	Bandipora District, Jammu and Kashmir State
35	74.7233333	34.4102778	~1800	?	India	Bandipora District, Jammu and Kashmir State
36	74.7188889	34.6469444	~2440	?	India	Bandipora District, Jammu and Kashmir State
37	74.3833333	34.0500000	2700	?	India	Baramulla District, Jammu and Kashmir State
38	74.7202778	34.0688889	1700	?	India	Srinagar District, Jammu and Kashmir State
39	74.7900000	34.0900000	1730	*chambensis*	India	Srinagar District, Jammu and Kashmir State
40	74.9469444	34.1244444	1895	?	India	Srinagar District, Jammu and Kashmir State
41	75.2913889	34.3002778	~2700	?	India	Ganderbal District, Jammu and Kashmir State
42	74.5199680	33.6299310	3490	?	India	Poonch District, Jammu and Kashmir State
43	74.0925000	33.7703000	~ 1000	?	India	Poonch District, Jammu and Kashmir State
44	74.9000000	33.8666667	1639	?	India	Pulwama District, Jammu and Kashmir State
45	75.2525000	33.9191667	2200	?	India	Anantnag District, Jammu and Kashmir State
46	75.2413889	34.1583333	2770	?	India	Anantnag District, Jammu and Kashmir State
47	75.2961111	34.0455556	2250	?	India	Anantnag District, Jammu and Kashmir State
48	75.3075000	34.0172222	2300	?	India	Anantnag District, Jammu and Kashmir State
49	75.2963889	34.0236111	2740	?	India	Anantnag District, Jammu and Kashmir State
50	75.3244444	34.0144444	2280	?	India	Anantnag District, Jammu and Kashmir State
51	75.3102778	34.0330556	2250	?	India	Anantnag District, Jammu and Kashmir State
52	75.2850000	33.8613889	1900	?	India	Anantnag District, Jammu and Kashmir State
53	75.7733333	33.3066667	1670	?	India	Kishtwar District, Jammu and Kashmir State
54	75.5524040	33.8299850	2550	?	India	Kishtwar District, Jammu and Kashmir State
55	75.5464806	33.1095583	980–1280	?	India	Doda District, Jammu and Kashmir State
56	75.6388167	33.0929111	1600–2300	?	India	Doda District, Jammu and Kashmir State

57	75.7084000	33.0890806	1700–2300	?	India	Doda District, Jammu and Kashmir State
58	75.7075000	32.9850000	1650	?	India	Doda District, Jammu and Kashmir State
59	75.3086111	32.8075000	800	?	India	Udhampur District, Jammu and Kashmir State
60	75.7519444	34.4325000	~ 3100	?	India	Kargil District, Ladakh
61	76.1493200	32.8390900	1738	*chambensis*	India	Chamba District, Himachal Pradesh State
62	76.1500000	32.9091667	1750	*himalayanus*	India	Chamba District, Himachal Pradesh State
63	76.1558333	32.8569444	1670	*himalayanus*	India	Chamba District, Himachal Pradesh State
64	76.1750000	32.8894444	1500	*himalayanus*	India	Chamba District, Himachal Pradesh State
65	76.1625000	32.8997222	2230	*himalayanus*	India	Chamba District, Himachal Pradesh State
66	76.0186111	32.5505556	2400	*himalayanus*	India	Chamba District, Himachal Pradesh State
67	75.9500000	32.5333333	1800–2050	?	India	Chamba District, Himachal Pradesh State
68	76.1277778	32.5569444	~1000	*chambensis*	India	Chamba District, Himachal Pradesh State
69	76.2507600	32.9387000	2543	*chambensis*	India	Chamba District, Himachal Pradesh State
70	75.9256230	32.4654900	1400	?	India	Chamba District, Himachal Pradesh State
71	76.3322880	32.2690940	2300	*himalayanus*	India	Kangra District, Himachal Pradesh State
72	76.5400000	32.1152778	~1300	*himalayanus*	India	Kangra District, Himachal Pradesh State
73	77.3916667	31.0791667	1320	*himalayanus*	India	Kullu District, Himachal Pradesh State
74	77.3861020	31.6517230	1650	*himalayanus*	India	Kullu District, Himachal Pradesh State
75	77.1653940	32.2369070	2660	*himalayanus*	India	Kullu District, Himachal Pradesh State
76	77.1716700	32.2420930	2240	*himalayanus*	India	Kullu District, Himachal Pradesh State
77	77.4824690	31.6712700	2090	*himalayanus*	India	Kullu District, Himachal Pradesh State
78	77.3956000	31.7443410	2150	*himalayanus*	India	Kullu District, Himachal Pradesh State
79	77.5115470	31.6857650	2440	*himalayanus*	India	Kullu District, Himachal Pradesh State
80	77.5115470	31.6857650	2000	*himalayanus*	India	Kullu District, Himachal Pradesh State
81	77.1927030	32.2704250	2320	*himalayanus*	India	Kullu District, Himachal Pradesh State
82	77.1899020	32.2733920	2240	*himalayanus*	India	Kullu District, Himachal Pradesh State
83	77.3288050	31.7655330	1960	*himalayanus*	India	Kullu District, Himachal Pradesh State
84	77.1892460	32.2431770	2050	*himalayanus*	India	Kullu District, Himachal Pradesh State
85	77.1696930	32.2550360	2060	*himalayanus*	India	Kullu District, Himachal Pradesh State
86	77.1757130	32.2450130	2120	*himalayanus*	India	Kullu District, Himachal Pradesh State
87	77.1899020	32.2733920	2250	*himalayanus*	India	Kullu District, Himachal Pradesh State
88	77.1833333	32.5833333	1300	*himalayanus*	India	Kullu District, Himachal Pradesh State
89	77.1738889	32.1380556	~1700	*himalayanus*	India	Kullu District, Himachal Pradesh State
90	77.1930556	32.1683333	1890	*himalayanus*	India	Kullu District, Himachal Pradesh State
91	77.1238889	32.1316667	2300	*himalayanus*	India	Kullu District, Himachal Pradesh State
92	77.4407833	31.9965444	2210	*himalayanus*	India	Kullu District, Himachal Pradesh State
93	78.2780556	31.5977778	3048	*himalayanus*	India	Kinnaur District, Himachal Pradesh State
94	78.3741472	31.3745083	3050–3300	*himalayanus*	India	Kinnaur District, Himachal Pradesh State
95	77.1722222	31.1033333	~2100	*himalayanus*	India	Shimla District, Himachal Pradesh State
96	77.6330556	31.4500000	1020	*himalayanus*	India	Shimla District, Himachal Pradesh State
97	77.2261111	31.1613889	1700	*himalayanus*	India	Shimla District, Himachal Pradesh State
98	77.7819444	31.4869444	2000	*himalayanus*	India	Shimla District, Himachal Pradesh State
99	77.7833333	31.5166667	~1900	*himalayanus*	India	Shimla District, Himachal Pradesh State
100	77.4991667	31.2483333	3048	*himalayanus*	India	Shimla District, Himachal Pradesh State
101	77.1394444	31.1180556	1982	*himalayanus*	India	Shimla District, Himachal Pradesh State
102	77.1736111	31.1088889	1960	*himalayanus*	India	Shimla District, Himachal Pradesh State
103	77.4036111	31.2166667	2743	*himalayanus*	India	Shimla District, Himachal Pradesh State
104	77.4527778	30.8372222	2438	*himalayanus*	India	Simaur District, Himachal Pradesh State
105	77.8066667	31.4933333	3048	*himalayanus*	India	Simaur District, Himachal Pradesh State
106	77.4550000	30.6144444	~ 900	*himalayanus*	India	Simaur District, Himachal Pradesh State
107	77.8713889	30.6991667	2150	*himalayanus*	India	Dehradun District, Uttarakhand State
108	78.0655556	30.4641667	1900	*himalayanus*	India	Dehradun District, Uttarakhand State
109	78.1017380	30.4580690	2250	*himalayanus*	India	Dehradun District, Uttarakhand State
110	78.0164611	30.4748000	2200	*himalayanus*	India	Dehradun District, Uttarakhand State
111	78.1038060	30.4540170	2130	*himalayanus*	India	Dehra Dun District, Uttarakhand State
112	78.0500000	30.3333333	670	*himalayanus*	India	Dehra Dun District, Uttarakhand State
113	77.5144444	30.2580556	850	*himalayanus*	India	Dehra Dun District, Uttarakhand State
114	78.6450000	30.9210000	3231	*himalayanus*	India	Uttarakhashi District, Uttarakhand State
115	78.9398400	30.9946950	3060	*himalayanus*	India	Uttarakhashi District, Uttarakhand State
116	78.3728330	31.1273150	2880	*himalayanus*	India	Uttarakhashi District, Uttarakhand State

117	78.4977778	30.8472222	2400	*himalayanus*	India	Uttarakhashi District, Uttarakhand State
118	78.2454787	31.0781204	2100	*himalayanus*	India	Uttarakhashi District, Uttarakhand State
119	78.3438889	31.1183333	2743	*himalayanus*	India	Uttarakhashi District, Uttarakhand State
120	78.2472370	30.4205440	2240	*himalayanus*	India	Theri Garhwal, Uttarakhand State
121	78.0977778	30.5858333	~3000	*himalayanus*	India	Theri Garhwal District, Uttarakhand State
122	79.2044940	30.6224140	2820	*himalayanus*	India	Rudraprayag District, Uttarakhand State
123	79.0752778	30.5202778	~1600	*himalayanus*	India	Rudraprayag District, Uttarakhand State
124	79.2052778	30.4913889	3050	*himalayanus*	India	Rudraparyag District, Uttarakhand State
125	79.2091667	30.4883333	~3300	*himalayanus*	India	Rudraparyag District, Uttarakhand State
126	77.9613889	30.9722222	3000	*himalayanus*	India	Chamoli District, Uttarakhand State
127	79.2111000	30.4789000	2850	*himalayanus*	India	Chamoli District, Uttarakhand State
128	79.5884370	30.6661400	2480	*himalayanus*	India	Chamoli District, Uttarakhand State
129	79.9105556	30.0644444	3200	*himalayanus*	India	Bageshwar District, Uttarakhand State
130	80.2771333	30.1951667	3535	*himalayanus*	India	Pithorargarh District, Uttarakhand State
131	80.0641667	29.7952778	1280	*himalayanus*	India	Pithorargarh District, Uttarakhand State
132	80.2373400	30.0715170	2150	*himalayanus*	India	Pithorargarh District, Uttarakhand State
133	80.1991111	30.0594722	3433	*himalayanus*	India	Pithorargarh District, Uttarakhand State
134	80.2462778	30.1089167	1794	*himalayanus*	India	Pithorargarh District, Uttarakhand State
135	80.4672222	30.4322222	2640	*himalayanus*	India	Pithorargarh District, Uttarakhand State
136	80.4152778	30.3605556	3430	*himalayanus*	India	Pithorargarh District, Uttarakhand State
137	80.2309660	30.0834590	2340	*himalayanus*	India	Pithorargarh District, Uttarakhand State
138	80.2444810	30.0982940	1920	*himalayanus*	India	Pithorargarh District, Uttarakhand State
139	80.2331440	30.0769200	2200	*himalayanus*	India	Pithorargarh District, Uttarakhand State
140	79.6800120	29.8297350	2043	*himalayanus*	India	Almora District, Uttarakhand State
141	79.4971030	29.6127390	1620	*himalayanus*	India	Almora District, Uttarakhand State
142	79.4511111	29.3963889	~2100	*himalayanus*	India	Naini Tal District, Uttarakhand State
143	79.5889130	29.4406050	2600	*himalayanus*	India	Naini Tal District, Uttarakhand State
144	79.4510000	29.4030000	2267	*himalayanus*	India	Naini Tal District, Uttarakhand State
145	79.4420000	29.3880000	2159	*himalayanus*	India	Naini Tal District, Uttarakhand State
146	79.4422222	29.3869444	2215	*himalayanus*	India	Naini Tal District, Uttarakhand State
147	79.6040860	29.4347970	2240	*himalayanus*	India	Naini Tal District, Uttarakhand State
148	79.5906660	29.4367560	2200	*himalayanus*	India	Naini Tal District, Uttarakhand State
149	79.6558240	29.4604130	2230	*himalayanus*	India	Naini Tal District, Uttarakhand State
150	79.5819390	29.4336390	2060	*himalayanus*	India	Naini Tal District, Uttarakhand State
151	81.7072222	30.0486111	2800	*nepalensis*	Nepal	Humla District, Karnali Province
152	81.7994860	29.9832990	3220	*nepalensis*	Nepal	Humla District, Karnali Province
153	82.1820000	29.2749000	2900	*nepalensis*	Nepal	Jumla District, Karnali Province
154	82.3505556	29.2147222	~ 2900	*nepalensis*	Nepal	Jumla District, Karnali Province
155	82.3683333	29.3294444	2740–3048	*nepalensis*	Nepal	Jumla District, Karnali Province
156	82.6027778	29.1211111	3048	*nepalensis*	Nepal	Dolpa District, Karnali Province
157	82.8322222	29.0283333	~ 2600	*nepalensis*	Nepal	Dolpa District, Karnali Province
158	83.6425000	28.6425000	2130	*nepalensis*	Nepal	Mustang District, Gandaki Zone
159	83.6469000	28.6050330	2105	*nepalensis*	Nepal	Mustang District, Gandaki Zone
160	83.6333333	28.6141667	2440	*nepalensis*	Nepal	Mustang District, Gandaki Zone
161	83.6000000	28.6166667	2500	*nepalensis*	Nepal	Mustang District, Gandaki Zone
162	83.6187830	28.6524330	2681	*nepalensis*	Nepal	Mustang District, Gandaki Zone
163	83.7000000	28.7666667	2700	*nepalensis*	Nepal	Mustang District, Gandaki Zone
164	83.6816667	28.7427778	2670	*nepalensis*	Nepal	Mustang District, Gandaki Zone
165	83.6448000	28.7087000	2400–2700	*nepalensis*	Nepal	Mustang District, Gandaki Zone
166	83.6648889	28.5621111	3242	*nepalensis*	Nepal	Mustang District, Gandaki Zone
167	83.0613889	28.4969444	2400–3000	*nepalensis*	Nepal	Baglung District, Gandaki Zone
168	83.2177778	28.6791667	3050	*nepalensis*	Nepal	Myagdi District, Gandaki Zone
169	83.2366667	28.5877778	2590	*nepalensis*	Nepal	Myagdi District, Gandaki Zone
170	84.3435500	28.5312570	2180	*nepalensis*	Nepal	Manang District, Gandaki Zone
171	84.3547220	28.5236830	1850	*nepalensis*	Nepal	Manang District, Gandaki Zone
172	84.3755556	28.4566667	1640	*nepalensis*	Nepal	Manang District, Gandaki Zone

Supplementary Table S2. List of *Gloydius* (*G.*) species and outgroups (outgr.) included in this study, with corresponding specimen identifiers (voucher, isolate, haplotype, or library) and GenBank accession numbers for each mitochondrial marker: 12S rRNA, 16S rRNA, cytochrome b (*cytb*), and NADH dehydrogenase subunit 4 (*nd4*).

Species	Identifier	*12S*	*16S*	*cytb*	*nd4*
G. angusticeps	SH2014 PLF5	-	-	KF997927	-
G. angusticeps	JS1306G1A	KY040540	KY040572	KY040626	KY040647
G. angusticeps	JS1306G1B	KY040541	KY040573	KY040627	KY040648
G. angusticeps	JS1507G5	KY040545	KY040577	KY040631	KY040652
G. blomhoffi	B524	AY352780	AY352719	AY352751	AY35281
G. blomhoffi (*brevicaudus*)	70Jingzhou	-	-	HQ528467	HQ528346
G. brevicaudus		EU913477	EU913477	EU913477	EU913477
G. brevicaudus	GP628	-	-	JQ687495	JQ687476
G. brevicaudus	DW004	JX661211	-	JX661198	JX661225
G. brevicaudus	DW005	JX661212	-	JX661199	JX661226
G. caraganus	426	MZ958021	MZ957012	MZ959165	MZ959158
G. caraganus	JS1610CR1	-	-	MF490455	MF490453
G. caraganus	JS1610CR2	-	-	MF490456	MF490454
G. caraganus	R290	-	-	-	KM078594
G. caucasicus	Gh26	-	-	MH370350	MH370357
G. caucasicus	Gh27	-	-	MH370351	MH370356
G. caucasicus	G68	-	-	MH370352	MH370354
G. caucasicus	G69	-	-	MH370353	MH370355
G. caucasicus	913	MZ958022	MZ957013	MZ959166	MZ959159
G. chambensis	17v13	-	OP518267	OP480161	OP407964
G. chambensis	18.13	-	OP518269	-	OP407966
G. chambensis	19.40	-	OP518272	OP480164	-
G. chambensis	17v20	OP508266	OP518268	OP480162	OP407965
G. chambensis	19.44	OP508268	OP518273	-	OP407970
G. chambensis	19.50	OP508269	-	OP480165	OP407971
G. chambensis	1854	-	-	-	OP407967
G. chambensis	1855	-	OP518270	-	OP407968
G. chambensis	NMW17079.1	PX280590	PX280554	-	-
G. changdaoensis	MT731652	MT731652	MT731652	MT731652	MT731652
G. cognatus	JSSD13109I1	-	-	KX063810	KX063783
G. cognatus	JSSD1505N6	-	-	KX063809	KX063782
G. cognatus	CIB93145	-	-	KX063811	KX063784
G. cognatus	BYEB1501IA	-	-	KX063812	KX063785
G. cognatus	JS15100005	-	-	_KX063814	KX063787
G. cognatus	QY224	KY040529	KY040561	KY040619	KY040640
G. cognatus	JSSD13109I3	KY040531	KY040563	KY040621	KY040642
G. cognatus	JS130947	KY040532	KY040564	KY040622	KY040643
G. halys	JSSD1508	-	-	-	KX063776
G. halys	SYNU1510151	-	-	KX063800	KX063773
G. halys	JSSD140M1	-	-	KX063804	KX063777
G. halys	DLG11	-	-	KX063805	KX063778
G. halys	DLG12	-	-	KX063806	KX063779
G. halys	DLHSKG50	-	-	KX063807	KX063780
G. halys	JS1607H9	-	-	KY040618	KY040639
G. halys	SYNU1301908	KY040526	KY040558	KX063802	KX063775
G. halys	1031	-	-	KX855938	-
G. halys	3517	-	-	KX855939	-
G. halys	3516	-	-	KX855940	-

G. halys	3514	-	-	KX855941	-
G. halys	3512	-	-	KX855942	-
G. halys	3511	-	-	KX855943	-
G. halys	3509	-	-	KX855944	-
G. halys	3504	-	-	KX855946	-
G. halys	3503	-	-	KX855947	-
G. halys	3502	-	-	KX855948	-
G. halys	3501	-	-	KX855949	-
G. halys	3500	-	-	KX855950	-
G. halys	JSSD1508X3	KY040527	KY040559	KX063803	-
G. hazarensis	PMNH4110	PX280593	PX280557	PX290126	PX290138
G. hazarensis	PMNH4240	PX280594	PX280558	PX290118	-
G. himalayanus		-	-	-	OP407973
G. himalayanus	BB87	-	-	PX290127	-
G. himalayanus	BB88	-	-	PX290128	-
G. himalayanus	HM_19.30	-	-	MZ959173	MZ959172
G. himalayanus		MK559438	MK559438	MK559438	MK559438
G. himalayanus	17v03	OP508270	OP518274	OP480166	OP407972
G. himalayanus	BMNH1946.1.19.64	PX280595	PX280559	-	-
G. himalayanus	ZMB2940	PX280596	PX280560	PX290129	PX290139
G. hindukushensis	NMW41993	PX280598	PX280561	PX290119	PX290133
G. hindukushensis	CUHC10088	PX280597	PX280562	PX290120	PX290134
G. hindukushensis	PMNH5150	-	PX280563	PX290121	PX290135
G. huangi	KIZ027654	MK227409	MK227412	MK227415	MK227418
G. huangi	CIB533422012	MW722961	MW721129	MZ355578	MW732035
G. intermedius (*cognatus*)*?*	GP188	-	-	JQ687488	JQ687469
G. intermedius		KM186844	KM186844	KM186844	KM186844
G. intermedius	NIBR0000625204	MW143075	MW143075	MW143075	MW143075
G. lateralis	JZ03	-	-	ON423419	ON423423
G. lateralis	JZ01	ON362225	ON362229	ON42341	ON423421
G. lateralis	JZ02	ON362226	ON362230	ON423418	ON423422
G. lateralis	JZ04	ON362228	ON362232	ON423420	ON423424
G. lijianlii	HS11047	-	-	KF997904	KF997956
G. lipipengi	JS1408G2	KY040542	KY040574	KY040628	KY040649
G. lipipengi (*swild; variegatus*)?	CD2	OK210581	OK184550	OK239652	OK23964
G. liupanensis	GP215	-	-	JQ687493	JQ687474
G. liupanensis	CHS083	MK065338	MK193903	MK201255	-
G. liupanensis	TC1	MZ958023	MZ957014	MZ959167	MZ959160
G. liupanensis	LP1	MZ958024	MZ957015	MZ959168	MZ959161
G. liupanensis	LP4	MZ958025	MZ957016	MZ959169	MZ959162
G. liupanensis	GP198	OP508271	OP518277	JQ687491	JQ687472
G. liupanensis	GP206	OP508272	-	JQ687492	JQ687473
G. monticola	JS1607DL1	KY040549	KY040581	KY040635	-
G. nepalensis	NME070555	PX280600	PX280564	PX290116	PX290132
G. nepalensis	NME.R.0544/07	PX280604	-	PX290117	-
G. nepalensis	RMNH20512	PX280599	PX280565	-	-
G. nepalensis	ZMB65611	PX280601	PX280566	PX290122	-
G. nepalensis	ZMB65612	PX280602	PX280567	PX290123	PX290136
G. nepalensis	ZMB65613	PX280603	PX280568	PX290124	PX290137
G. qinlingensis (*rubromaculatus*)*?*	SAFS2019001	MW690593	MW690593	MW690593	MW690593
G. qinlingensis		MF523224	MF523224	MF523224	MF523224
G. qinlingensis	JS1505QL1	KY040534	KY040566	KY040623	KY040644
G. qinlingensis	GP197	OP508273	-	JQ687490	JQ687471
G. rickmersi	ES2015	-	-	-	KM078592

G. rickmersi	ES2015_G64	-	-	-	KM096379
G. rickmersi	ZMB80360 (DJ11387)	-	-	PX290125	-
G. rubromaculatus	IOZ002317	KY040546	KY040578	KY040632	KY040653
G. rubromaculatus	JS1607Y5	KY040548	KY040580	KY040634	KY040655
G. changdaoensis	JSSD1408Z1	-	-	KX063821	KX063794
G. changdaoensis	JSSD1510C1	-	-	KX063823	KX063796
G. saxatilis	GP191	-	-	JQ687489	JQ687470
G. saxatilis	GP1328	-	OP518276	JQ687502	JQ687483
G. saxatilis	QS002	JX661216	-	JX661205	JX661228
G. saxatilis	unknown (Heilongjiang)	KM434236	KM434236	KM434236	KM434236
G. shedaoensis	GP1100	-	-	JQ687498	JQ687479
G. shedaoensis	GP1110	-	-	JQ687499	JQ687480
G. shedaoensis	GP1116	-	-	JQ687500	JQ687481
G. shedaoensis	HS110682	-	-	KF997910	-
G. shedaoensis	HS11062	-	-	KF997911	KF997971
G. shedaoensis	HS11067	-	-	KF997912	KF997970
G. shedaoensis	1119	KT726957	KT726957	KT726957	KT726957
G. shedaoensis	B856	OP508274	OP518279	OP480167	OP407975
G. shedaoensis (*qianshanensis*)	JS150722	KY040524	KY040556	KY040617	KY040638
G. shedaoensis	JSSD1110D2	-	-	KX063819	KX063792
G. stejnegeri	SYNU1510134(_S1)	-	-	KX063816	KX063789 (SYNU1510134_S1)
G. stejnegeri	JSSD1409S3	-	-	KX063817	KX063790
G. stejnegeri	JSSD1508S4	-	-	KX063818	KX063791
G. stejnegeri	SYNU1510145 (_53)	KY040538	KY040570	KX063815	KX063788 (SYNU1510145_53)
G. stejnegeri	JS151054	KY040539	KY040571	KY040625	KY040646
G. strauchi	JS1309G3	KY040543	KY040575	KY040629	KY040650
G. strauchi	JS1508G4	KY040544	KY040576	KY040630	KY040651
G. strauchi	GS190617	-	-	MN199353	MN199354
G. swild	GP175	-	-	JQ687487	JQ687468
G. swild (*variegatus*)?	GR1	OK210582	OK184551	OK239653	OK239648
G. tsushimaensis		JN870186	JN870196	JN870203	JN870211
G. ussuriensis	unknown (Heilongjiang)	KP262412	KP262412	KP262412	KP262412
G. ussuriensis	unknown (Heilongjiang)	NC_026553	NC_026553	NC_026553	NC_026553
G. ussuriensis	GP1326	OP508275	OP518280	JQ687501	JQ687482
G. lipipengi (*variegatus*)	CIB121712	PQ732981	PQ732983	PQ726910	PQ72691
outgr.: *Agkistrodon contortrix*	FTB1712	MK313326	MK313353	MK313557	MK313438
outgr.: *Azemiops feae*		NC_030781	NC_030781	NC_030781	NC_030781
outgr.: *Crotalus adamanteus*		MH626511	MH626511	MH626511	MH626511
outgr.: *Crotalus adamanteus*	CLP_4	-	-	AY223605	-
outgr.: *Crotalus durissus*	1097	-	-	EU624302	AY704885
outgr.: *Crotalus ravus*	UTA live	MN527047	-	AY223609	AY223647
outgr.: *Crotalus tigris*		-	-	AY223606	AF156574
outgr.: *Daboia russellii*		EU913478	EU913478	EU913478	EU913478
outgr.: *Daboia mauritanica*		-	-	KJ415304	EU624229
outgr.: *Montivipera wagneri*	wg3, CLP-568	-	-	KX168770	JN870213
outgr.: *Macrovipera lebetina*	lb63	-	-	KJ415301	-
outgr.: *Macrovipera schweizeri*	1643	EU624262	AJ275768	AJ275715	-
outgr.: *Macrovipera schweizeri*		MH717075	MH717075	MH717075	MH717075
outgr.: *Montivipera albizona*	bg10	-	-	KX168728	-
outgr.: *Montivipera xanthina*	xt9	-	-	KX168811	-
outgr.: *Protobothrops mucrosquamatus*		NC_021412	NC_021412	NC_021412	NC_021412
outgr.: *Ovophis monticola*	CAS215050	DQ305416	DQ305439	DQ305462	DQ305480
outgr.: *Ovophis okinavensis*		NC_007397	NC_007397	NC_007397	NC_007397

outgr.: *Porthidium arcosae*	750	EU624241	GQ372871	AF292575	AF292613
outgr.: *Porthidium lansbergii rozei*	787	EU624242	GQ372870	AY713375	AF393623
outgr.: *Porthidium nasutum*	ROM_42214	KX694567	KX694658	KX694838	U41887
outgr.: *Sistru catenatus*	Moody502	-	-	AY223610	AY223648
outgr.: *Sistrurus miliarius*		MK330875	MK330875	MK330875	MK330875
outgr.: *Sistru miliarius*	UTA_live	-	-	AY223611	U41889
outgr.: *Trimeresurus gracilis*	NTNUB 200515	DQ305415	DQ305438	DQ305461	DQ305479
outgr.: *Trimeresurus tibetanus*	B258	AY352776	AY352715	AY352749	AY352810
outgr.: *Vipera ammodytes ammodytes*	haplotype_bSR3	-	-	DQ186513	EU624232
outgr.: *Vipera aspis*	CLP-573/Vas13/E072	JN870190	-	KR153577	JX649606
outgr.: *Vipera aspis*	15VA083	-	-	MT156829	MT157027
outgr.: *Vipera berus*	haplotype J47 (Vb410C)	-	-	DQ186079	-
outgr.: *Vipera berus*	Vber416	-	-	FR72710	FR727036
outgr.: *Vipera eriwanensis*	haplotype C26/N20, lib. EriArm	-	-	FR727090	FR727021
outgr.: *Vipera graeca*		-	-	LT220960	HG940665
outgr.: *Vipera graeca*	N1	-	-	HG94067	HG940665
outgr.: *Vipera kaznakovi*	K001	-	-	FR727103	FR727034
outgr.: *Vipera latastei*	E001	-	-	AY321094	JX649540
outgr.: *Vipera latastei*	P156/ 14VL020	KY762037	KY762066	MG875549	JX649564
outgr.: *Vipera lotievi*	library Lot1	-	-	FR727097	FR727030
outgr.: *Vipera renardi*	haplotype C9/N6, library Ren28	-	-	FR727101	FR72703
outgr.: *Vipera seoanei*	library S101	-	-	DQ186030	FR727035
outgr.: *Vipera ursinii*	PE144	-	-	FM955005	FR726956
outgr.: *Vipera (b.) walser*	It1_L14724vb	-	-	KX357735	-

Supplementary Figure S1. Maximum-likelihood tree generated with IQ-TREE 2 from 2,693 bp of concatenated mitochondrial sequences. The tree is split across panels, with the right panel showing the continuation. Node support was estimated from 1,000 replicates, and values are shown only for nodes with SH-aLRT > 80%. Node labels report support in the following order: SH-aLRT (Shimodaira–Hasegawa approximate likelihood ratio test), LBP (local bootstrap probability), aBayes (approximate Bayesian support), and UFBoot (ultrafast bootstrap). LBP and aBayes represent single-branch support measures. Major Himalayan *Gloydius* lineages are highlighted.

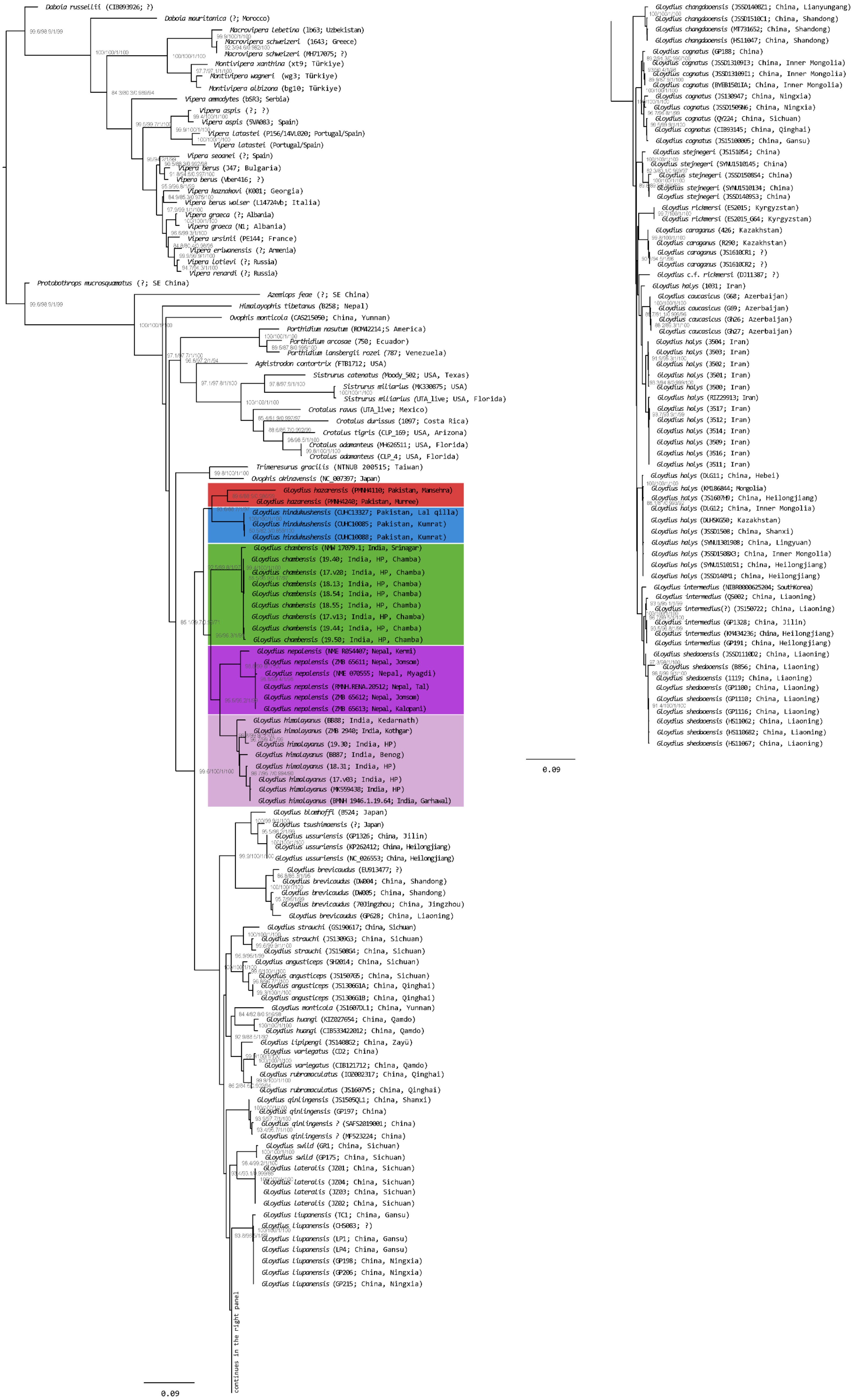

Supplementary Fig. S3. Maxent response curves for the study species showing the marginal effect of each environmental predictor on the predicted probability of occurrence. Curves represent the mean response across 100 replicate model runs (red), with ±1 standard deviation shown in blue. Each predictor is varied individually while all other predictors are held at their mean sample values.

G. hazarensis

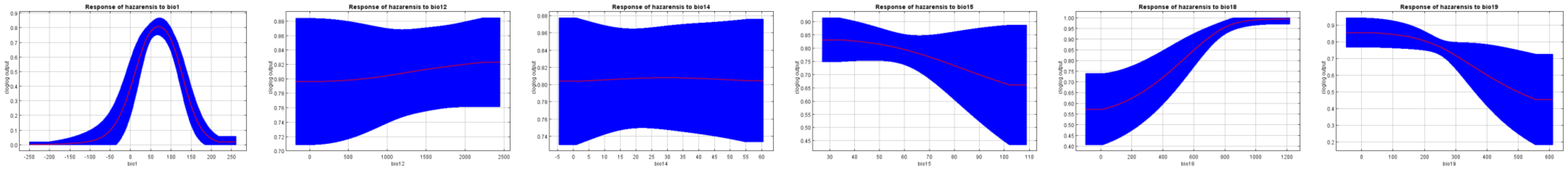

G. himalayanus

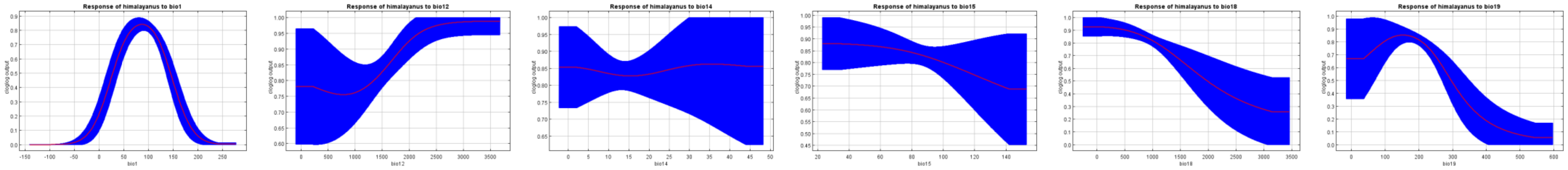

G. hindukushensis

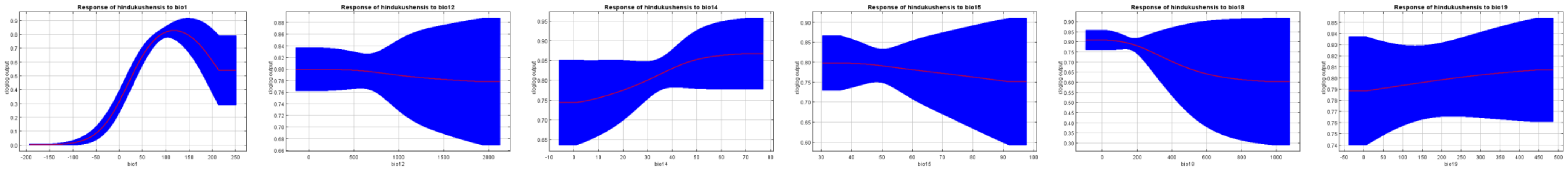

G. nepalensis

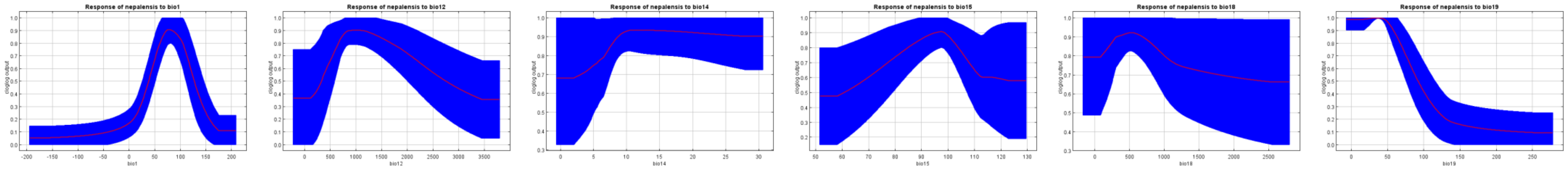

Supplement: Supplementary file 1 [file ECE3-16-e74238-s001.zip › 3_SupportingInformation1.pdf]
